# Supplementary material for: A non-canonical repressor function of JUN restrains YAP activity and liver cancer growth
Source: EMBO J. 2024 Aug 29;43(20):4578–603. doi: 10.1038/s44318-024-00188-0 (PMC11480203; doi:10.1038/s44318-024-00188-0)
Supplement: Supplementary file 1 — Appendix [file 44318_2024_188_MOESM1_ESM.pdf]

APPENDIX

A non-canonical repressor function of JUN restrains YAP activity and liver cancer growth

Yuliya Kurlishchuk, Anita Cindric Vranesic, Marco Jessen, Alexandra Kipping, Christin Ritter, KyungMok Kim, Paul Cramer, Björn von Eyss

Table of Contents

Appendix Figure S1: Proliferation rate of MCF10A cells is not affected by JUN overexpression 2

Appendix Figure S2: Efficient JUN depletion and metabolic labeling of mRNA in MCF10A$^{JUN\text{-}AID\text{-}V5}$ cells 3

Appendix Figure S3: JUN depends on NCOR1/2 to repress YAP target genes 4

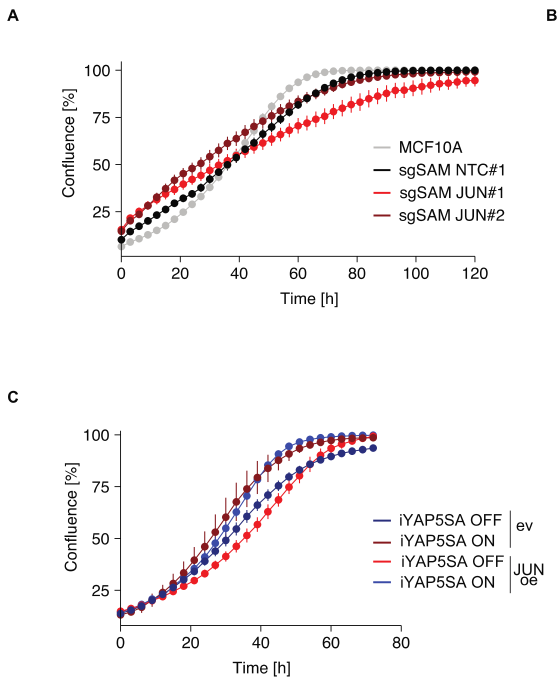

Appendix Figure S1: Proliferation rate of MCF10A cells is not affected by JUN overexpression

A.-C. Incucyte growth curves for the indicated MCF10A cell lines. Where appropriate, YAP5SA expression was induced with 1 µg/ml doxycycline or cells were treated with ethanol as a solvent control. ev = empty vector, oe = overexpression.

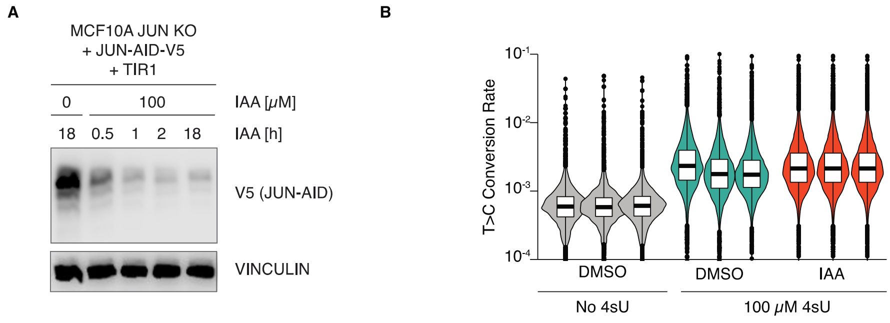

Appendix Figure S2: Efficient JUN depletion and metabolic labeling of mRNA in MCF10A$^{JUN\text{-}AID\text{-}V5}$ cells

A. Immunoblot from MCF10A$^{JUN\text{-}AID\text{-}V5}$ cells after IAA addition. MCF10A JUN knockout cells expressing a JUN allele fused to a V5-tagged auxin-inducible degron (JUN-AID-V5) were treated with 100 µM IAA for indicated times and subjected to immunoblotting. Addition of IAA efficiently induced JUN degradation. As control, cells were treated with DMSO. Vinculin was used as loading control. IAA = indole-3-acetic acid.

B. Violin plots of thymine-to-cytosine (T>C) conversion rates in SLAM-Seq of MCF10A$^{JUN\text{-}AID\text{-}V5}$ cells. Cells were subjected to 100 µM 4sU pulse prior to RNA isolation. 4sU-labeled RNA was alkylated and subjected to 3'-end RNA-Sequencing. The plot illustrates efficient T>C conversion upon 4sU labeling, when compared to cells not treated with 4sU. IAA = indole-3-acetic acid, 4sU = 4-thiouridine.

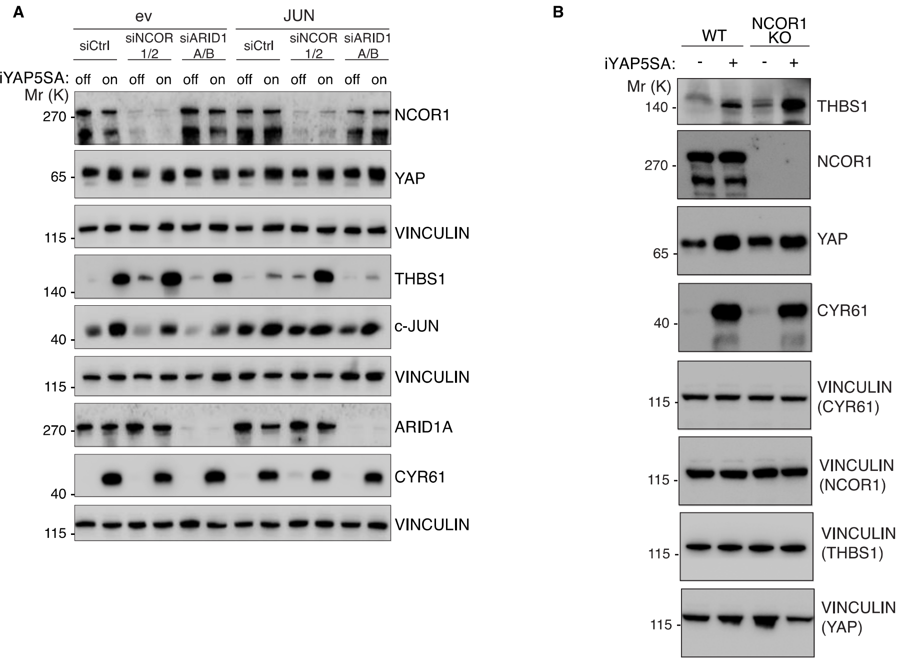

Appendix Figure S3: JUN depends on NCOR1/2 to repress YAP target genes

A. Immunoblot as shown in Fig. 6A, including all VINCULIN blots as loading controls. ev = empty vector.
B. Immunoblot as shown in Fig. 6B, including all VINCULIN blots as loading controls.
