# Supplementary figures and images for: A non-canonical repressor function of JUN restrains YAP activity and liver cancer growth
Source: EMBO J. 2024 Aug 29;43(20):4578–603. doi: 10.1038/s44318-024-00188-0 (PMC11480203; doi:10.1038/s44318-024-00188-0)

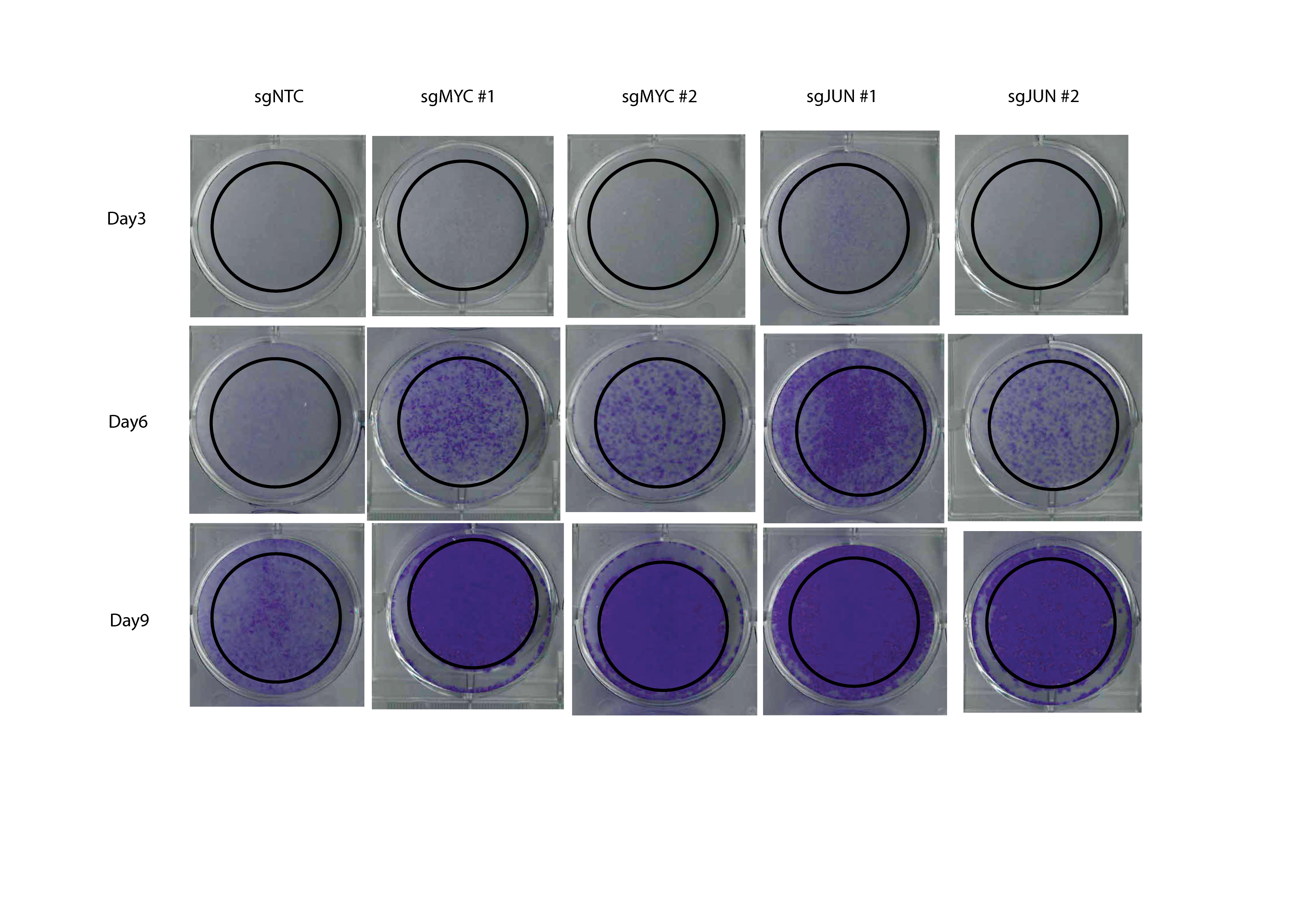

Supplement: Supplementary file 8 — Source data Fig. 1 [file 44318_2024_188_MOESM8_ESM.zip › Figure_1/1H/Figure_1H.tif]

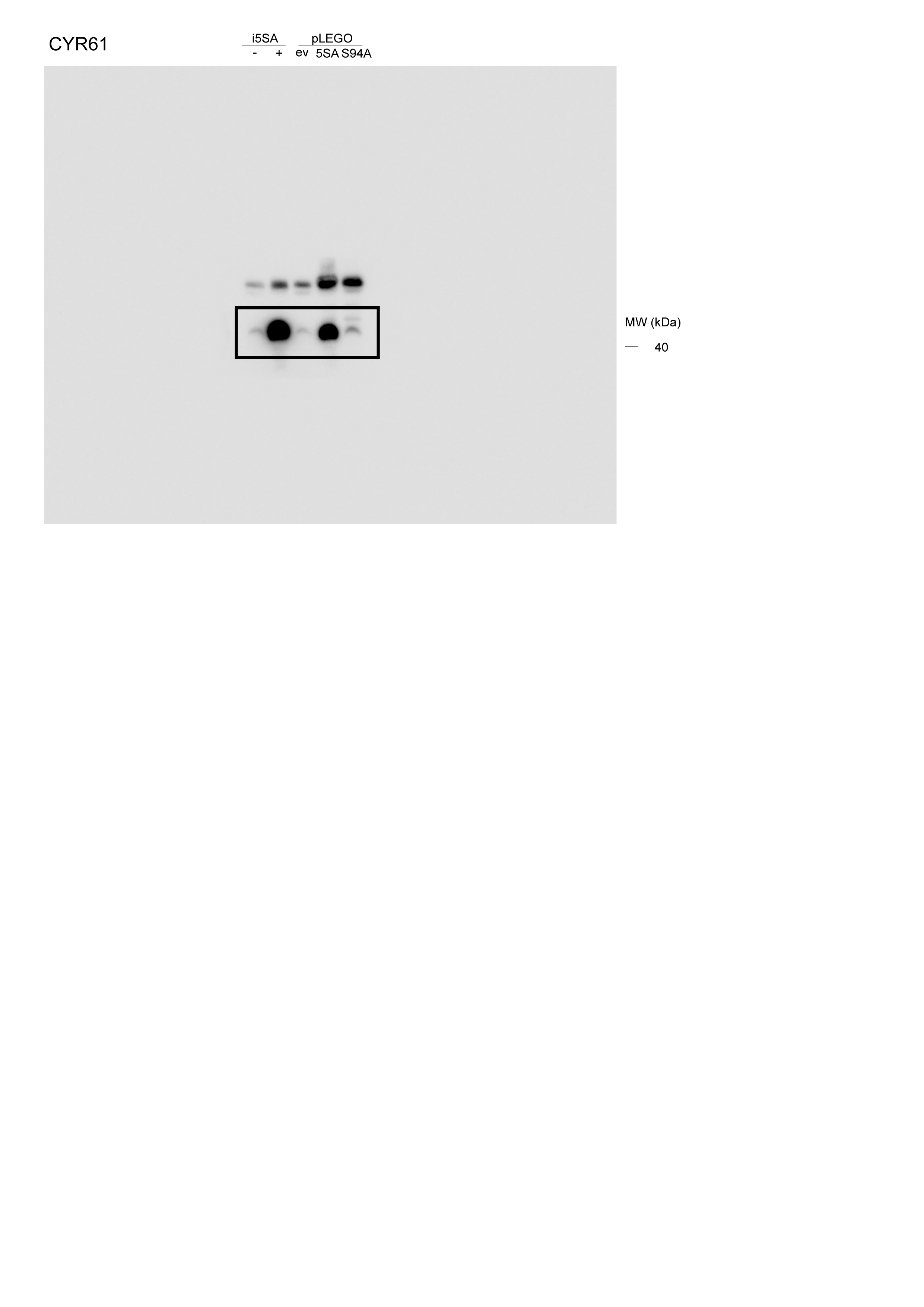

Supplement: Supplementary file 8 — Source data Fig. 1 [file 44318_2024_188_MOESM8_ESM.zip › Figure_1/1A/Figure_1A_CYR61.tif]

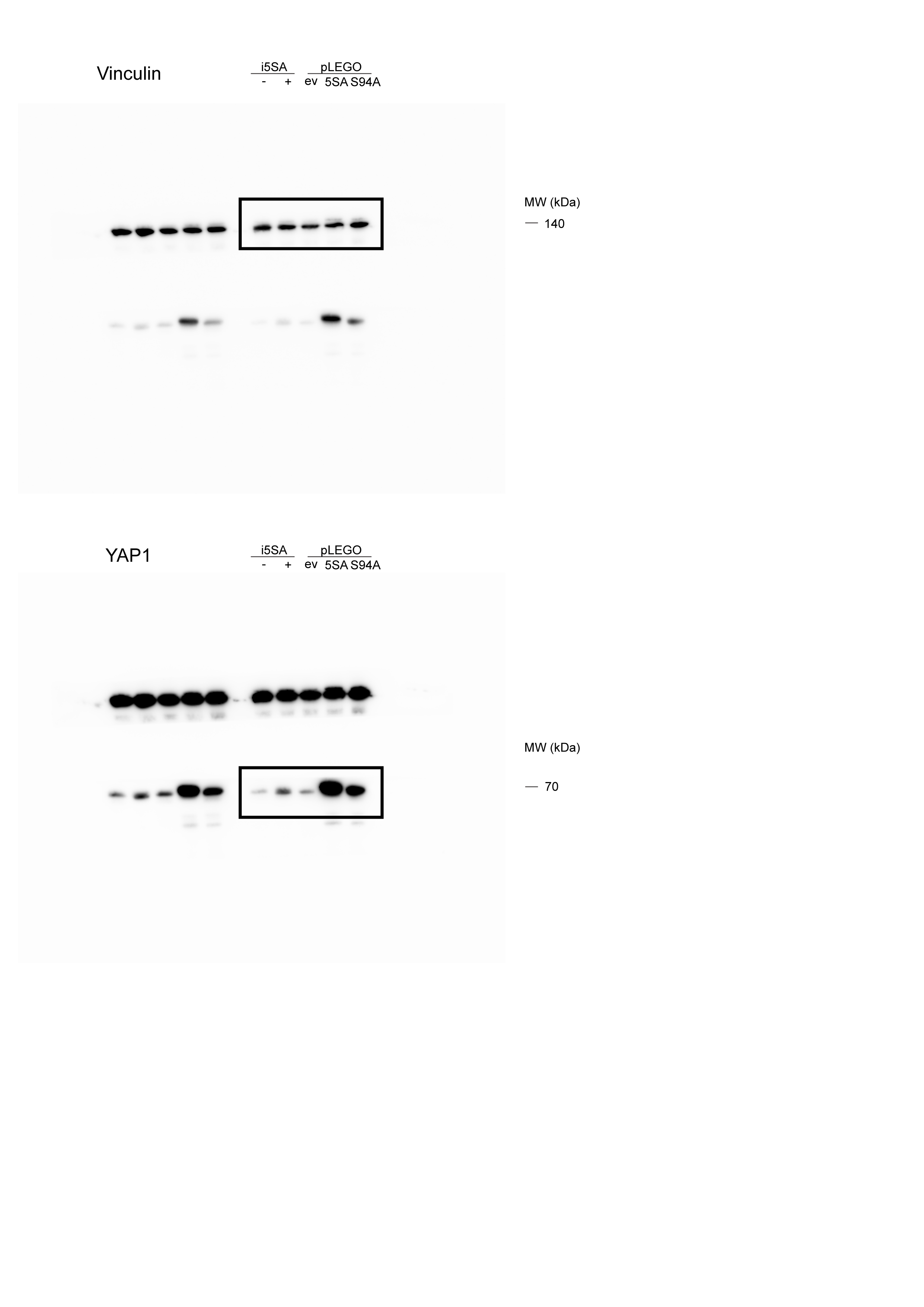

Supplement: Supplementary file 8 — Source data Fig. 1 [file 44318_2024_188_MOESM8_ESM.zip › Figure_1/1A/Figure_1A_YAP_VINCULIN.tif]

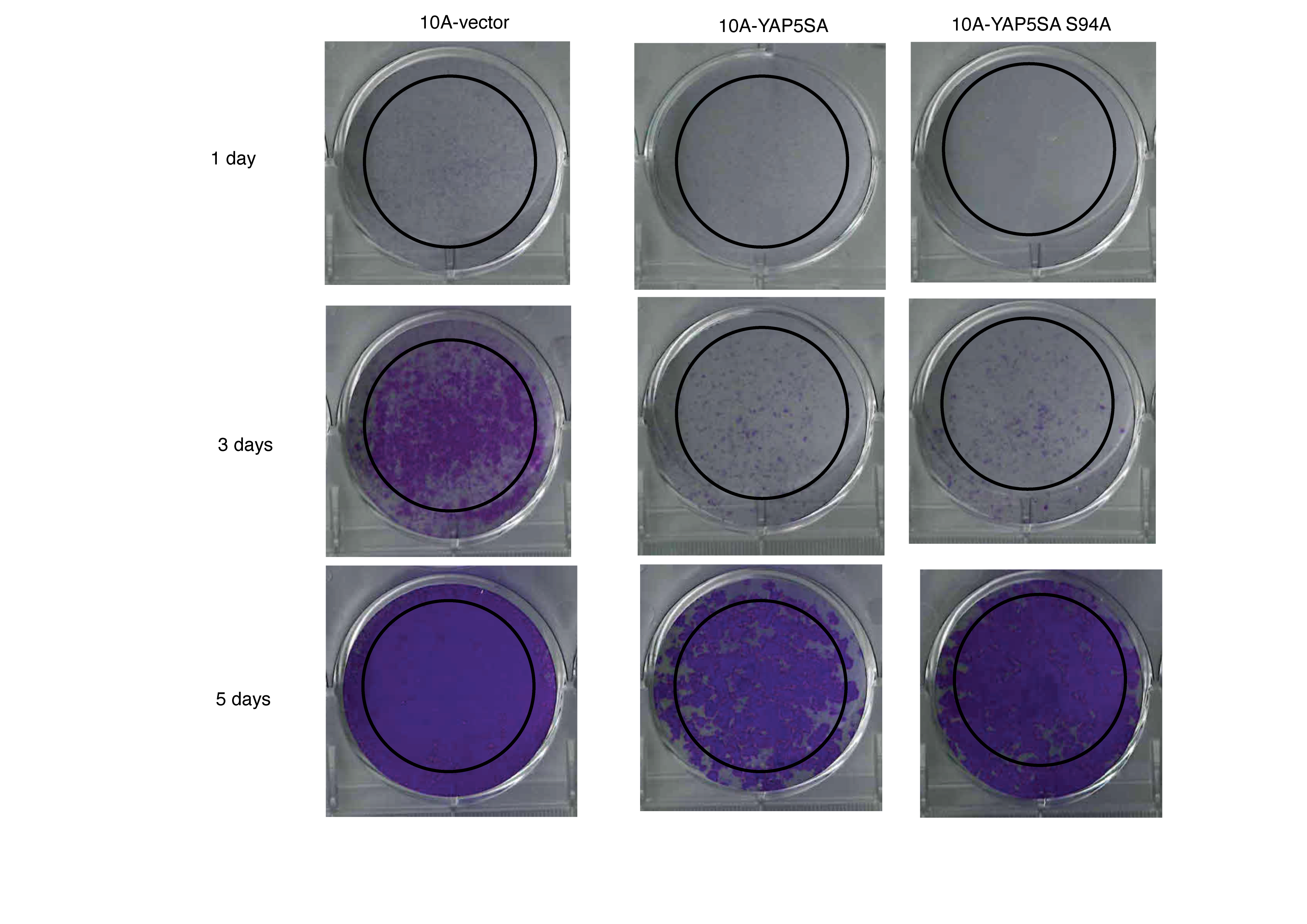

Supplement: Supplementary file 8 — Source data Fig. 1 [file 44318_2024_188_MOESM8_ESM.zip › Figure_1/1D/Figure_1D.tif]

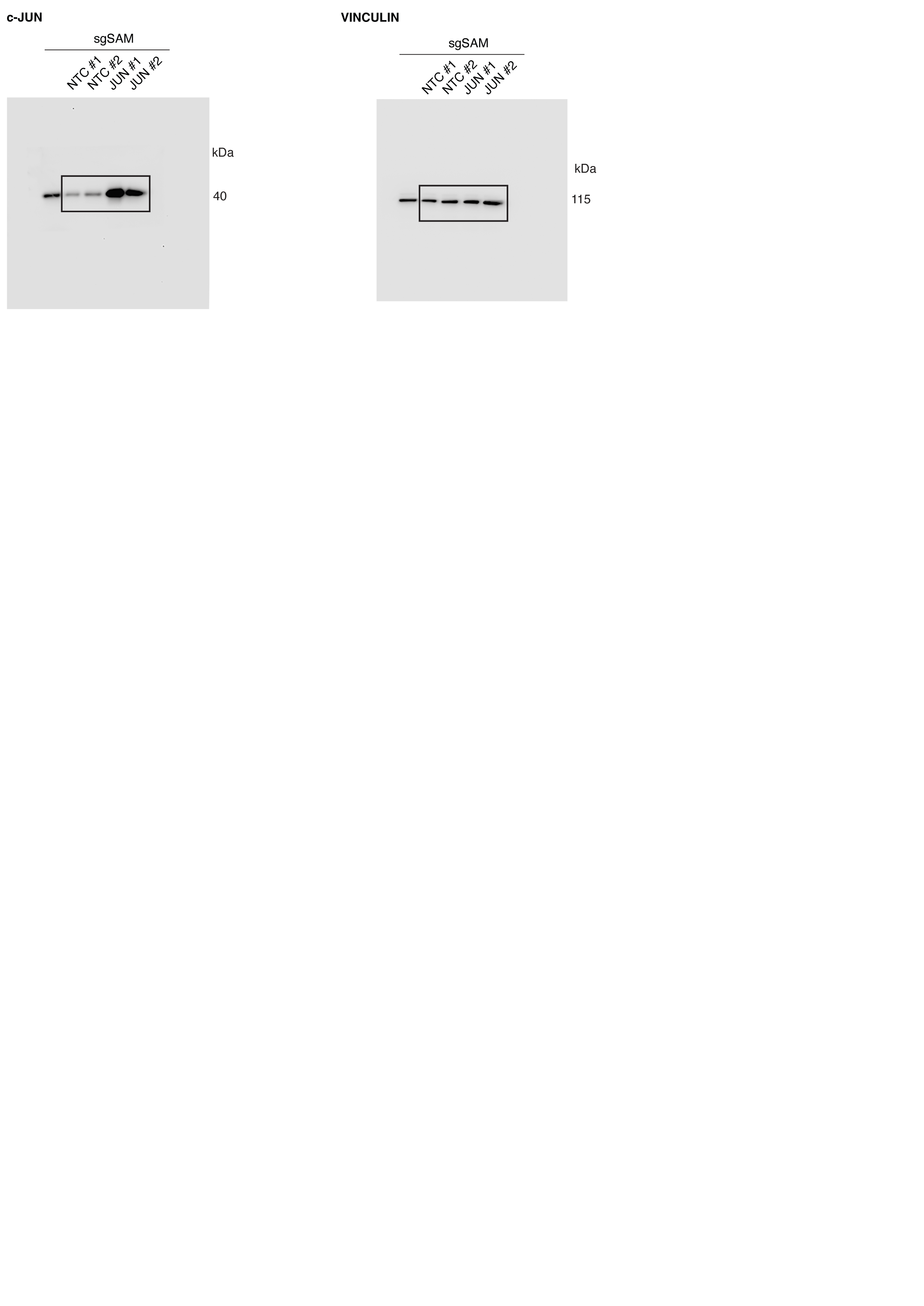

Supplement: Supplementary file 8 — Source data Fig. 1 [file 44318_2024_188_MOESM8_ESM.zip › Figure_1/1J/Figure_1J.tif]

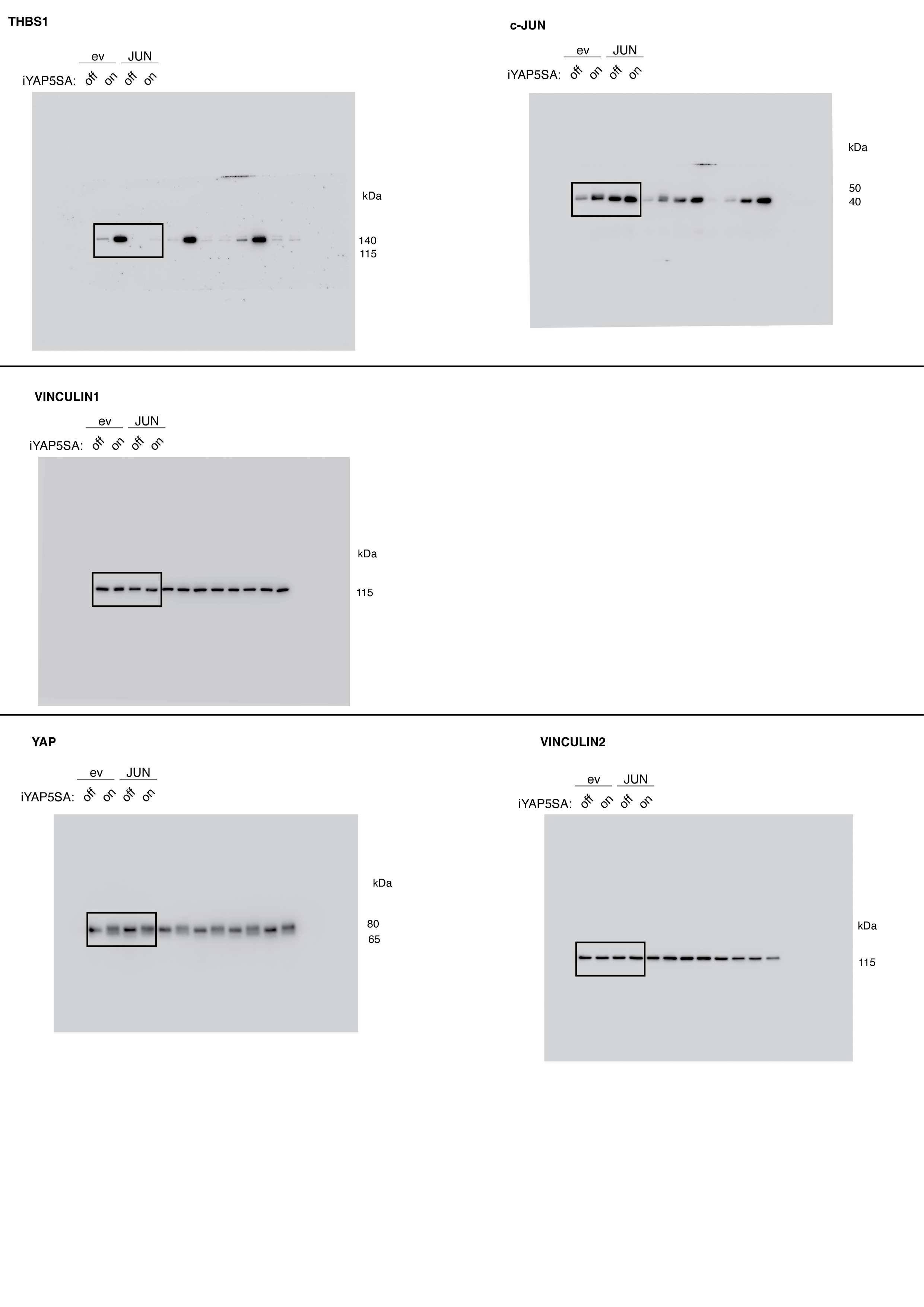

Supplement: Supplementary file 9 — Source data Fig. 2 [file 44318_2024_188_MOESM9_ESM.zip › Figure_2/2I/Figure_2I.tif]

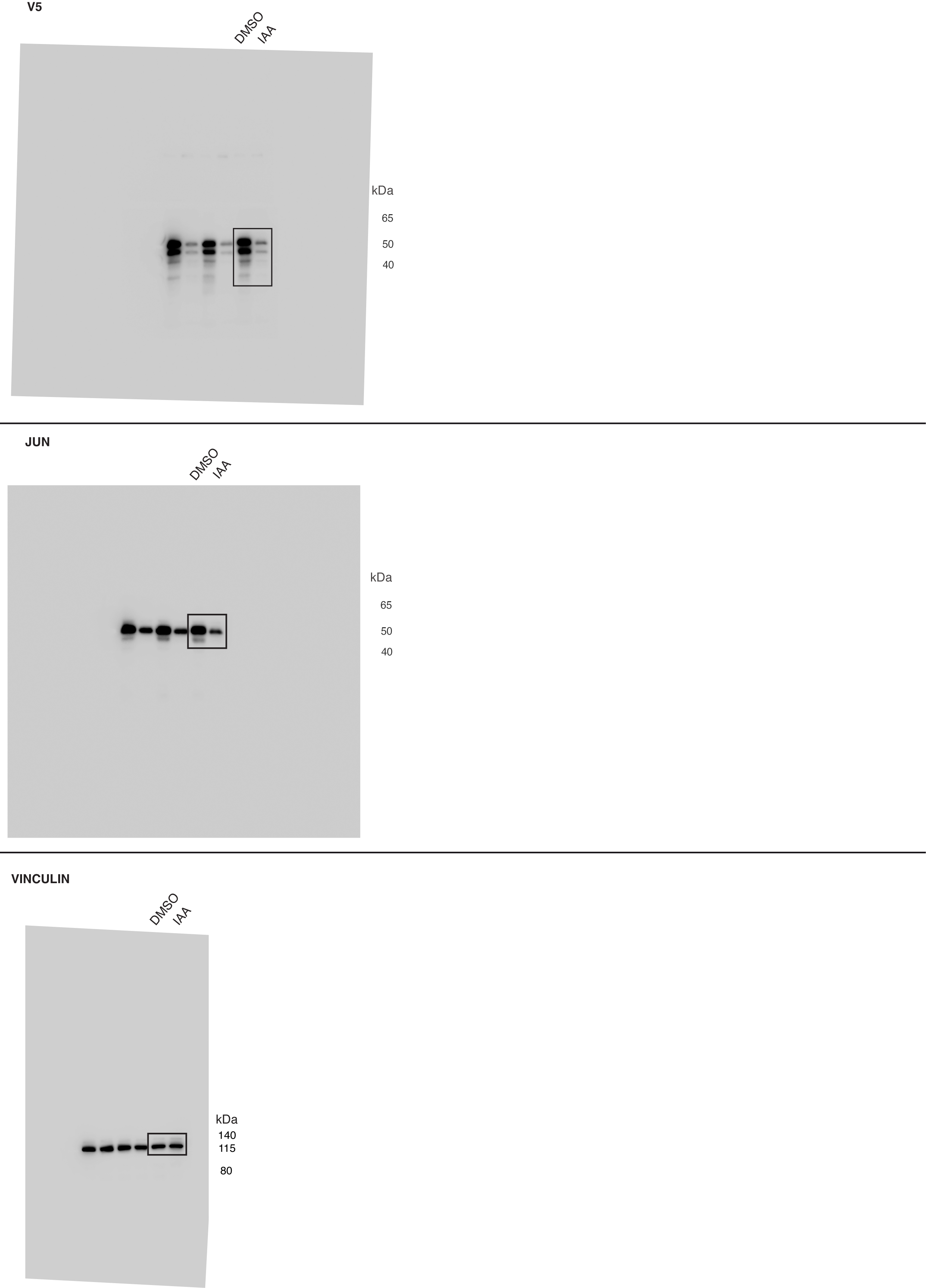

Supplement: Supplementary file 9 — Source data Fig. 2 [file 44318_2024_188_MOESM9_ESM.zip › Figure_2/2O/Figure_2O.tif]

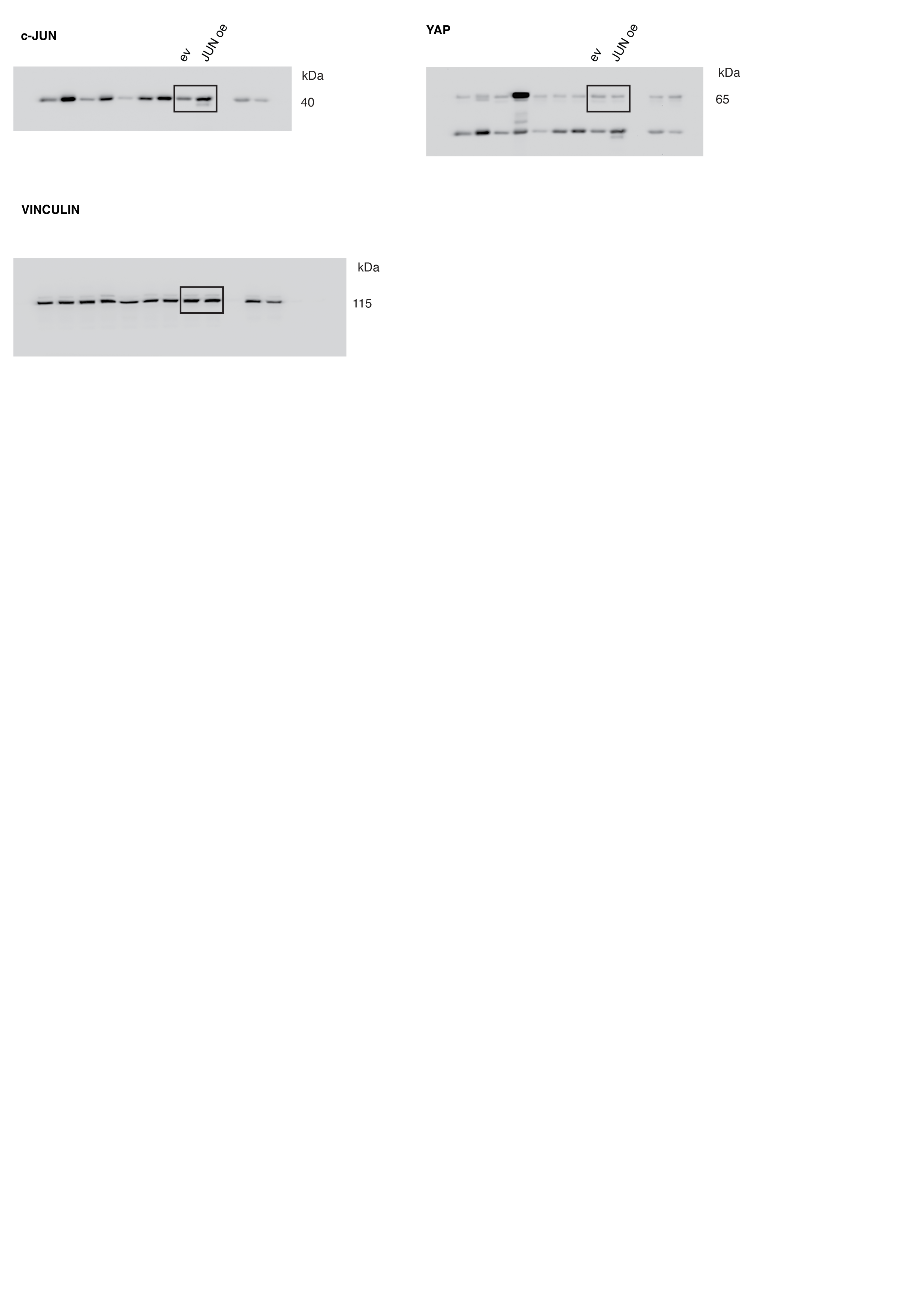

Supplement: Supplementary file 9 — Source data Fig. 2 [file 44318_2024_188_MOESM9_ESM.zip › Figure_2/2C/Figure_2C.tif]

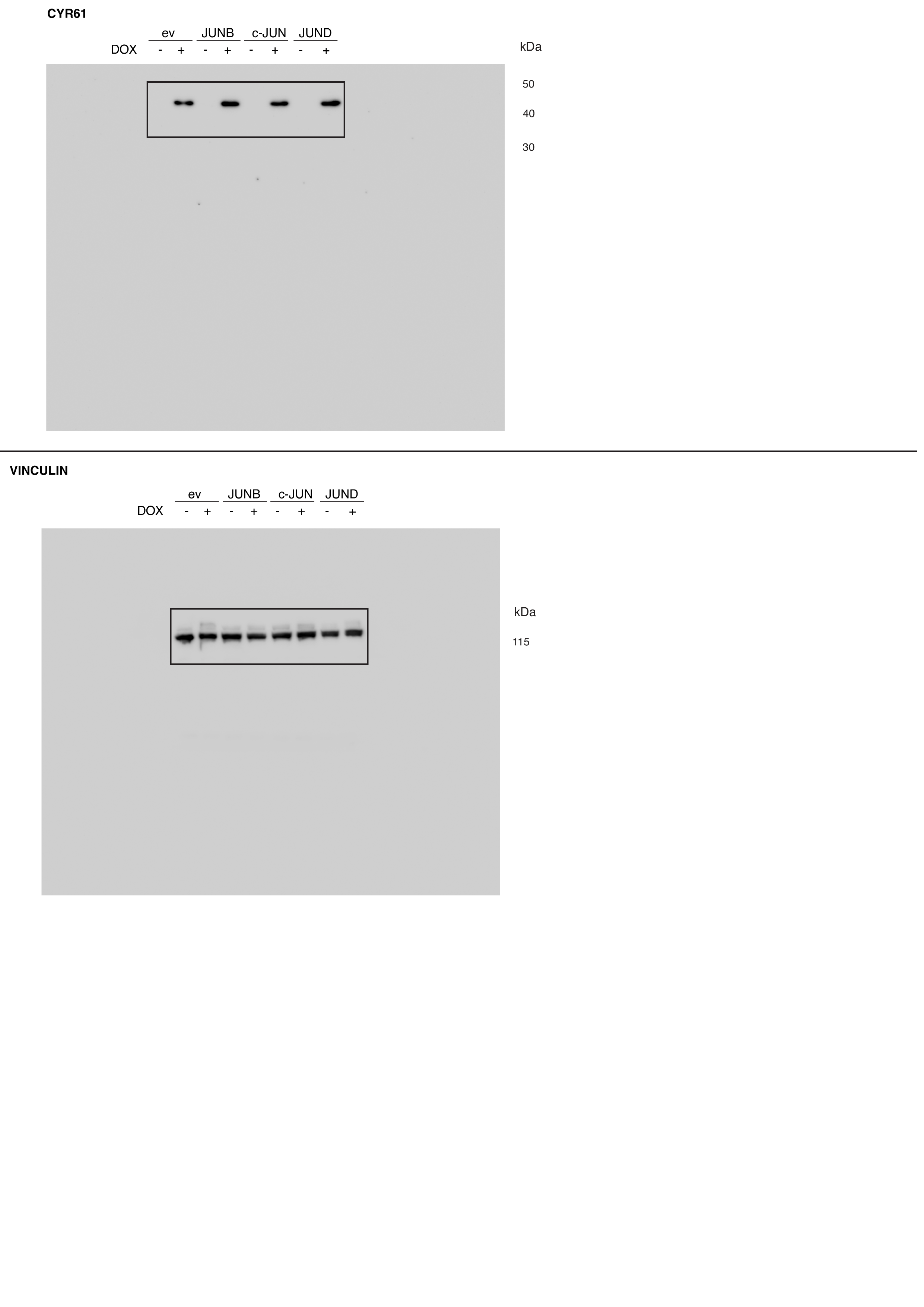

Supplement: Supplementary file 9 — Source data Fig. 2 [file 44318_2024_188_MOESM9_ESM.zip › Figure_2/2J/Figure_2J_CYR61_VINCULIN.tif]

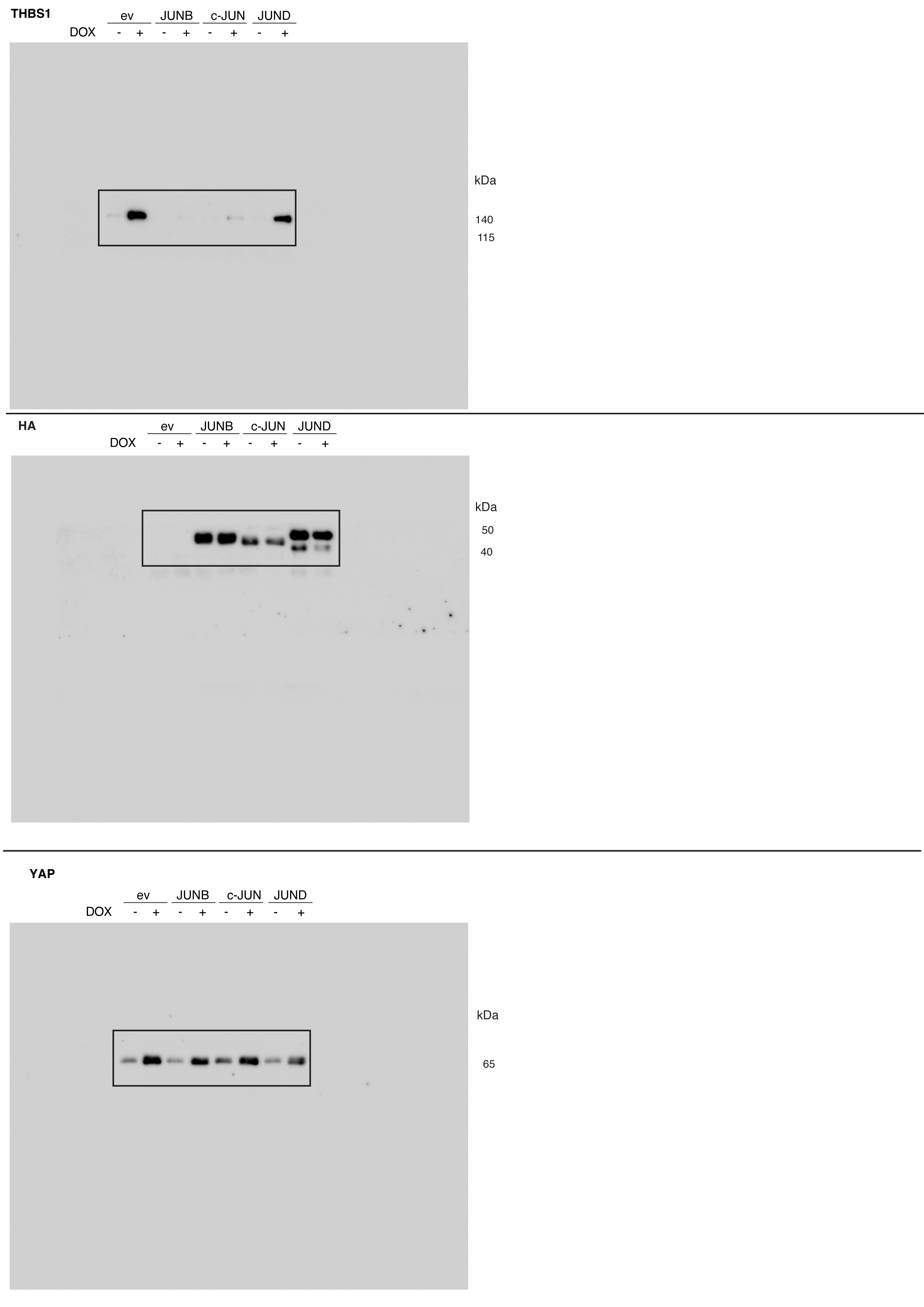

Supplement: Supplementary file 9 — Source data Fig. 2 [file 44318_2024_188_MOESM9_ESM.zip › Figure_2/2J/Figure_2J_THBS1_HA_YAP.tif]

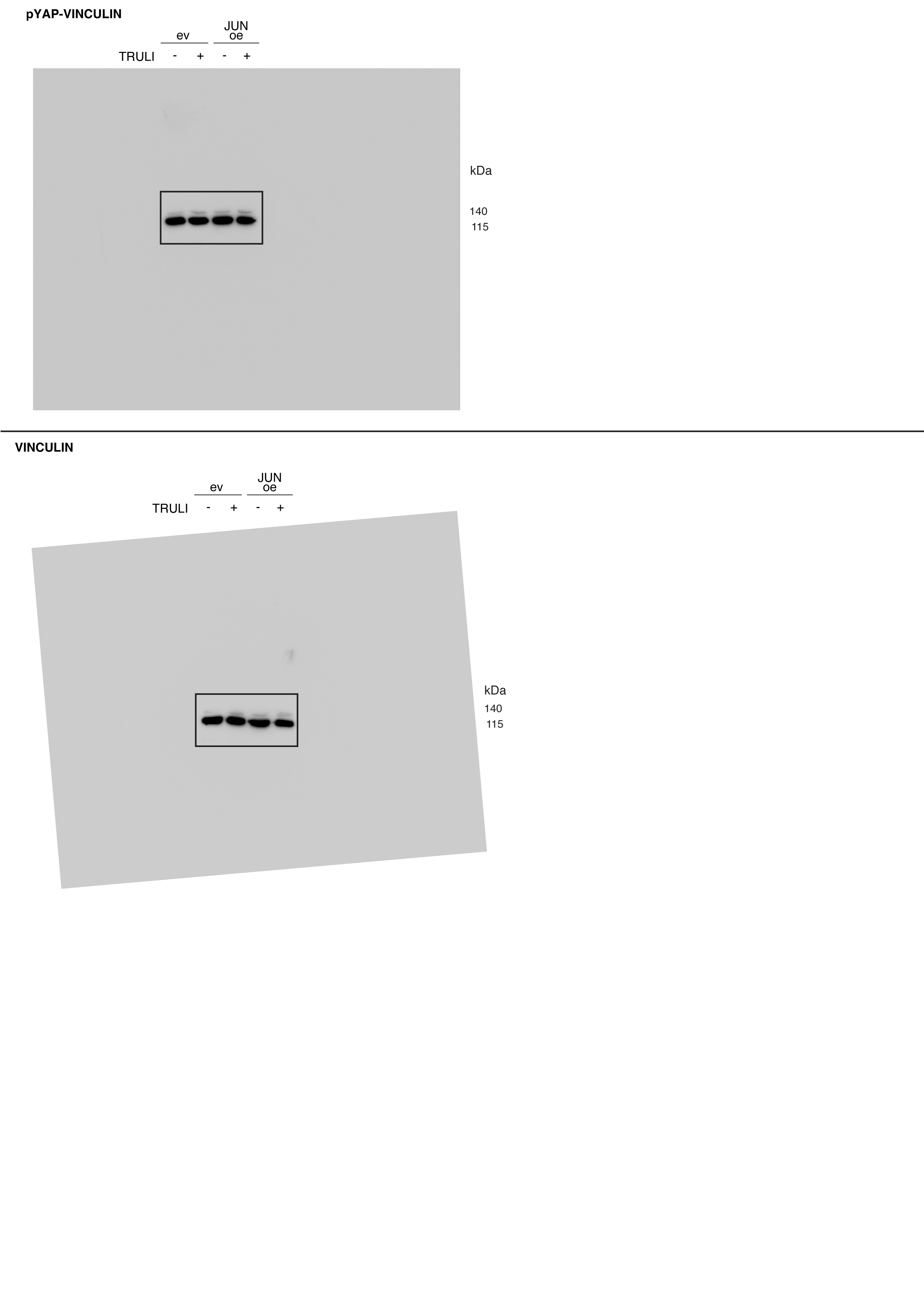

Supplement: Supplementary file 9 — Source data Fig. 2 [file 44318_2024_188_MOESM9_ESM.zip › Figure_2/2L/Figure_2L_pYAP-VINCULIN_VINCULIN.tif]

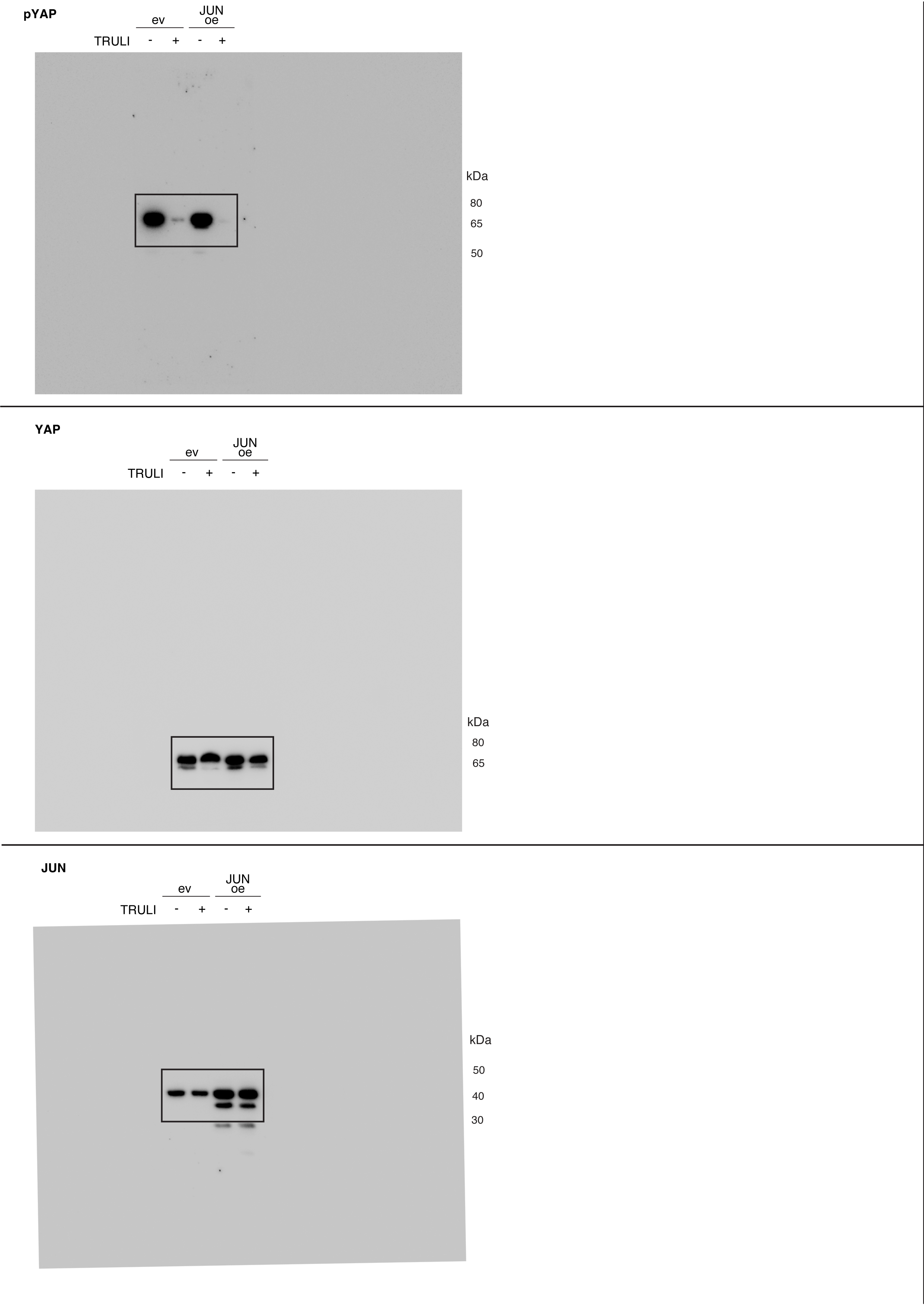

Supplement: Supplementary file 9 — Source data Fig. 2 [file 44318_2024_188_MOESM9_ESM.zip › Figure_2/2L/Figure_2L_pYAP_YAP_JUN.tif]

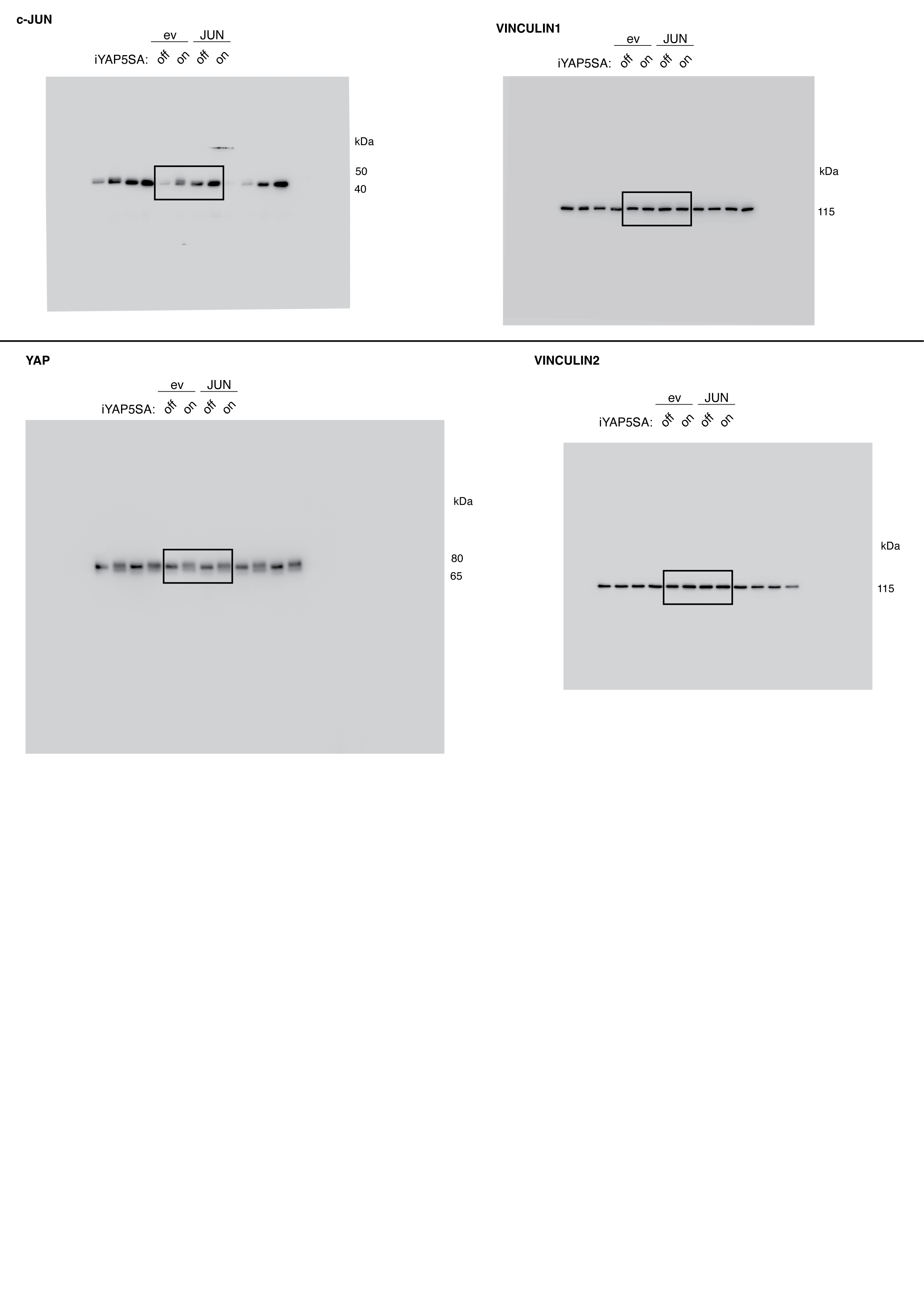

Supplement: Supplementary file 9 — Source data Fig. 2 [file 44318_2024_188_MOESM9_ESM.zip › Figure_2/2E/Figure_2E.tif]

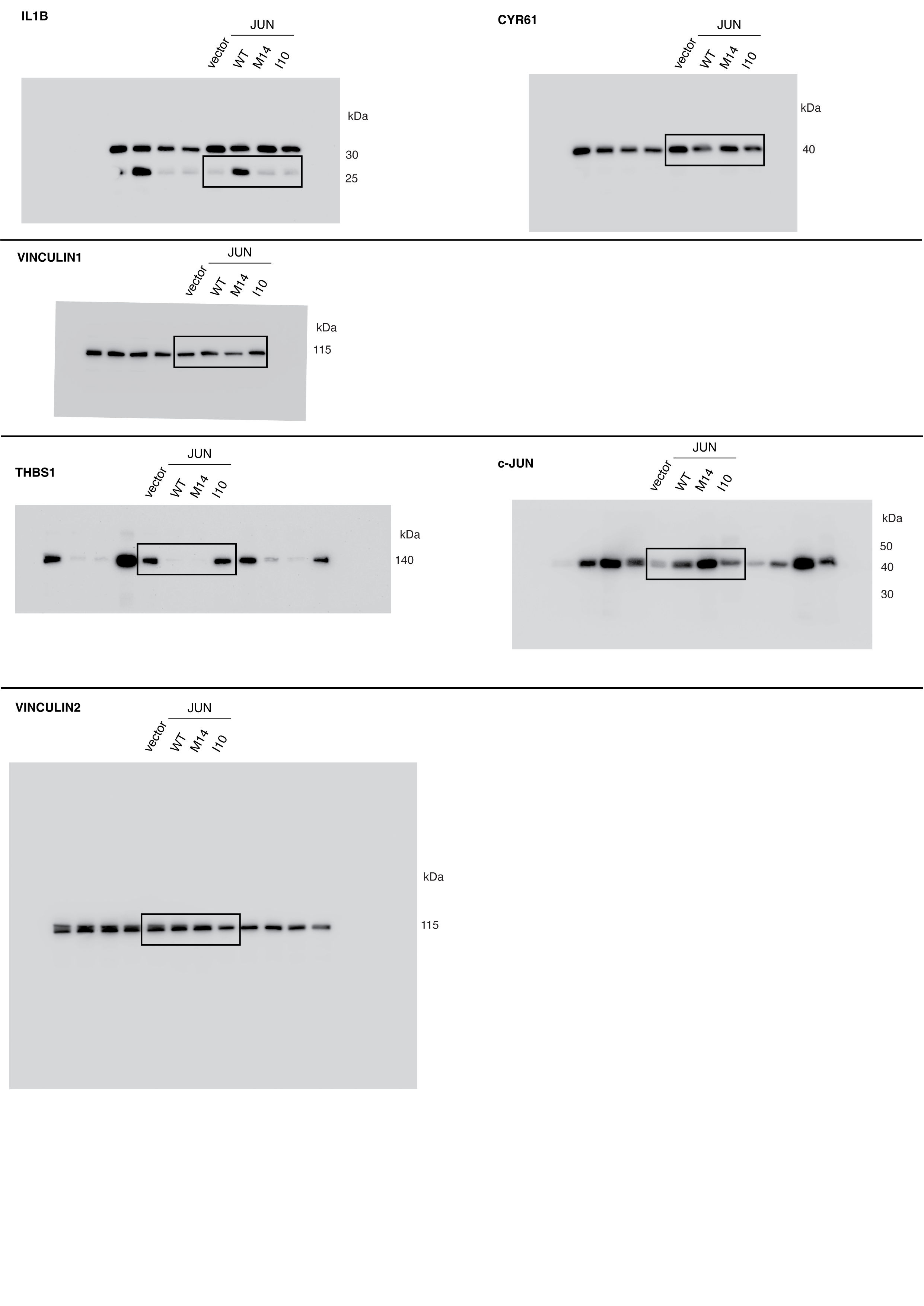

Supplement: Supplementary file 10 — Source data Fig. 4 [file 44318_2024_188_MOESM10_ESM.zip › Figure_4/4D/Figure_4D.tif]

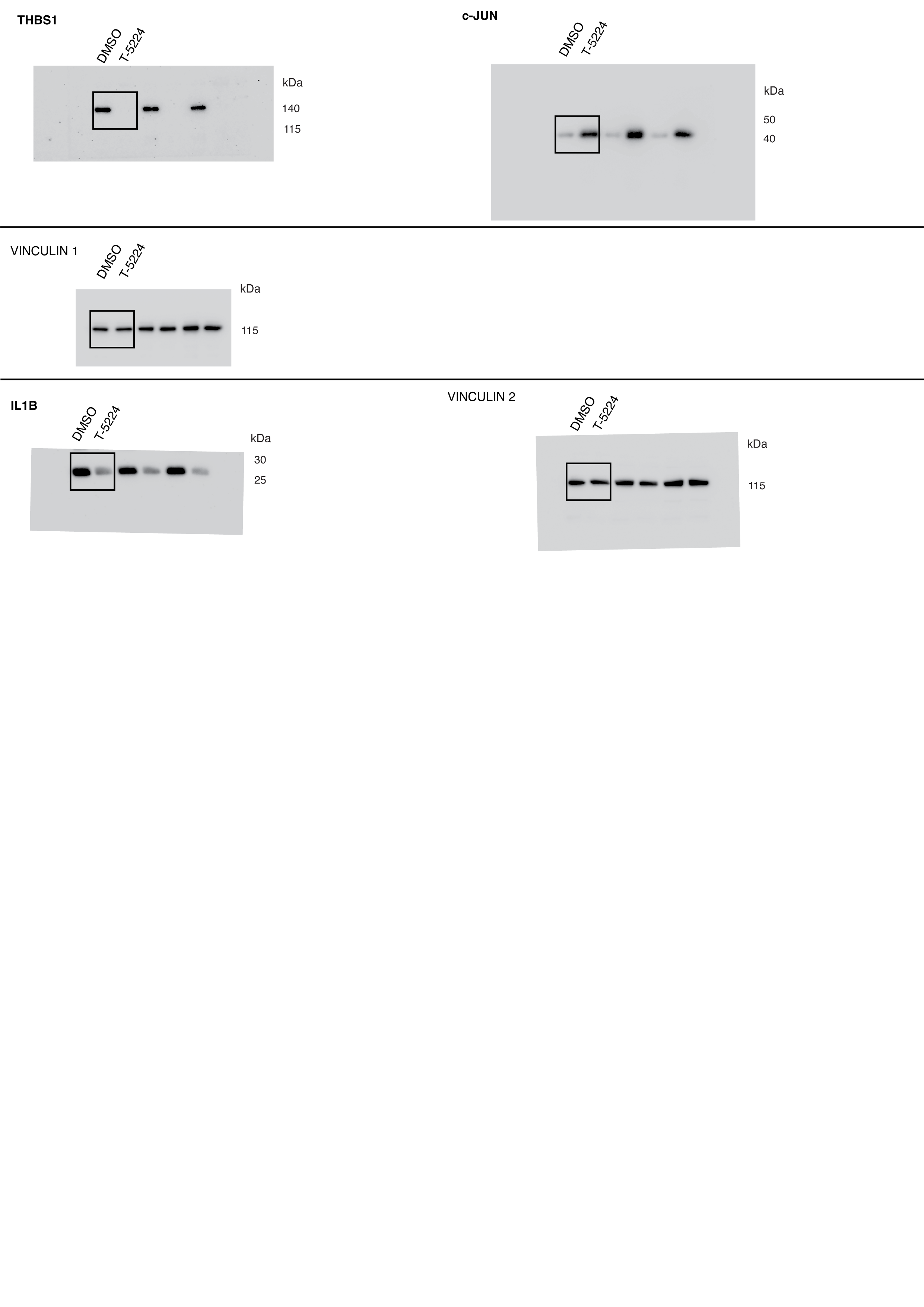

Supplement: Supplementary file 10 — Source data Fig. 4 [file 44318_2024_188_MOESM10_ESM.zip › Figure_4/4A/Figure_4A.tif]

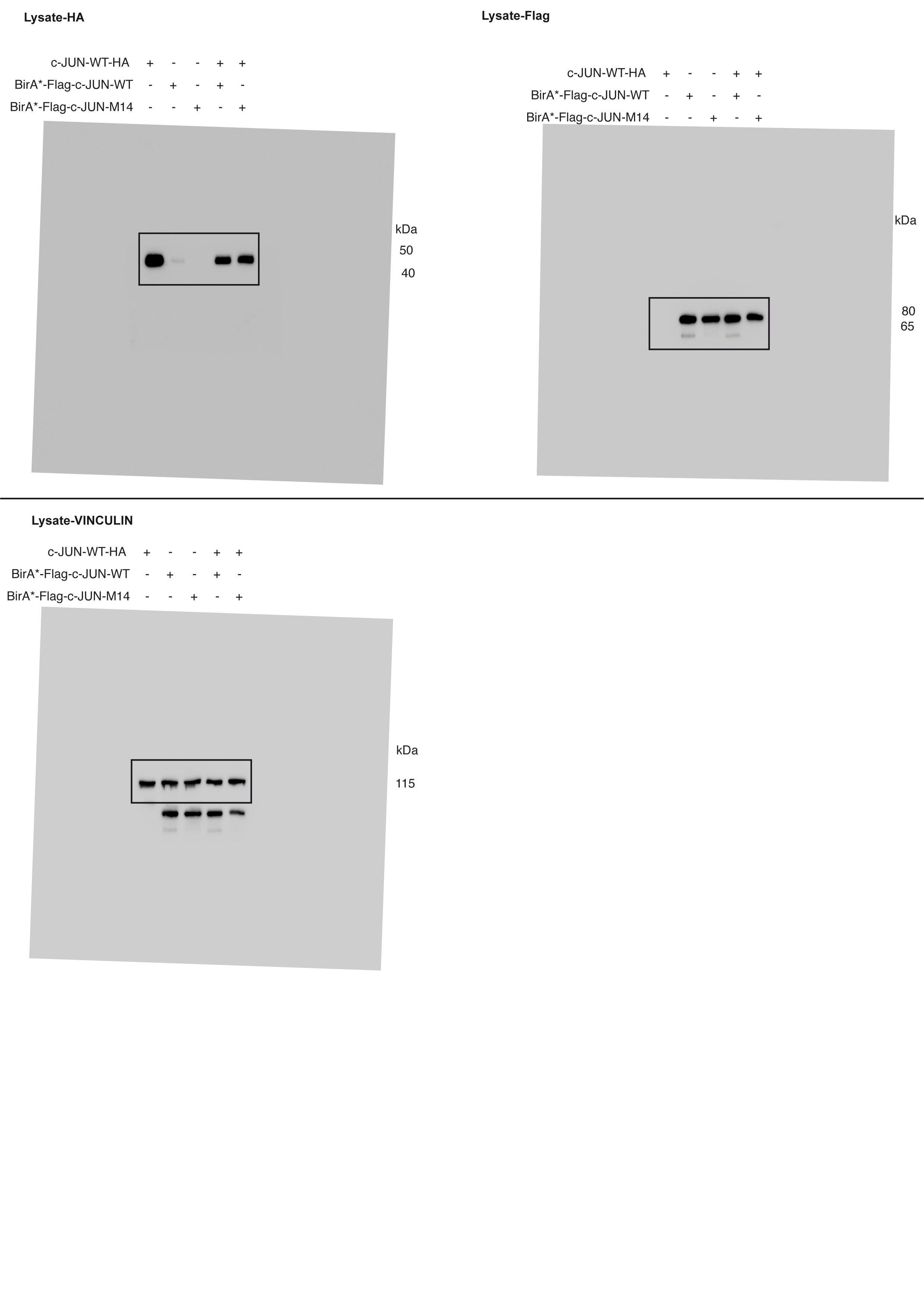

Supplement: Supplementary file 11 — Source data Fig. 5 [file 44318_2024_188_MOESM11_ESM.zip › Figure_5/5I/Figure_5I_Lysate.tif]

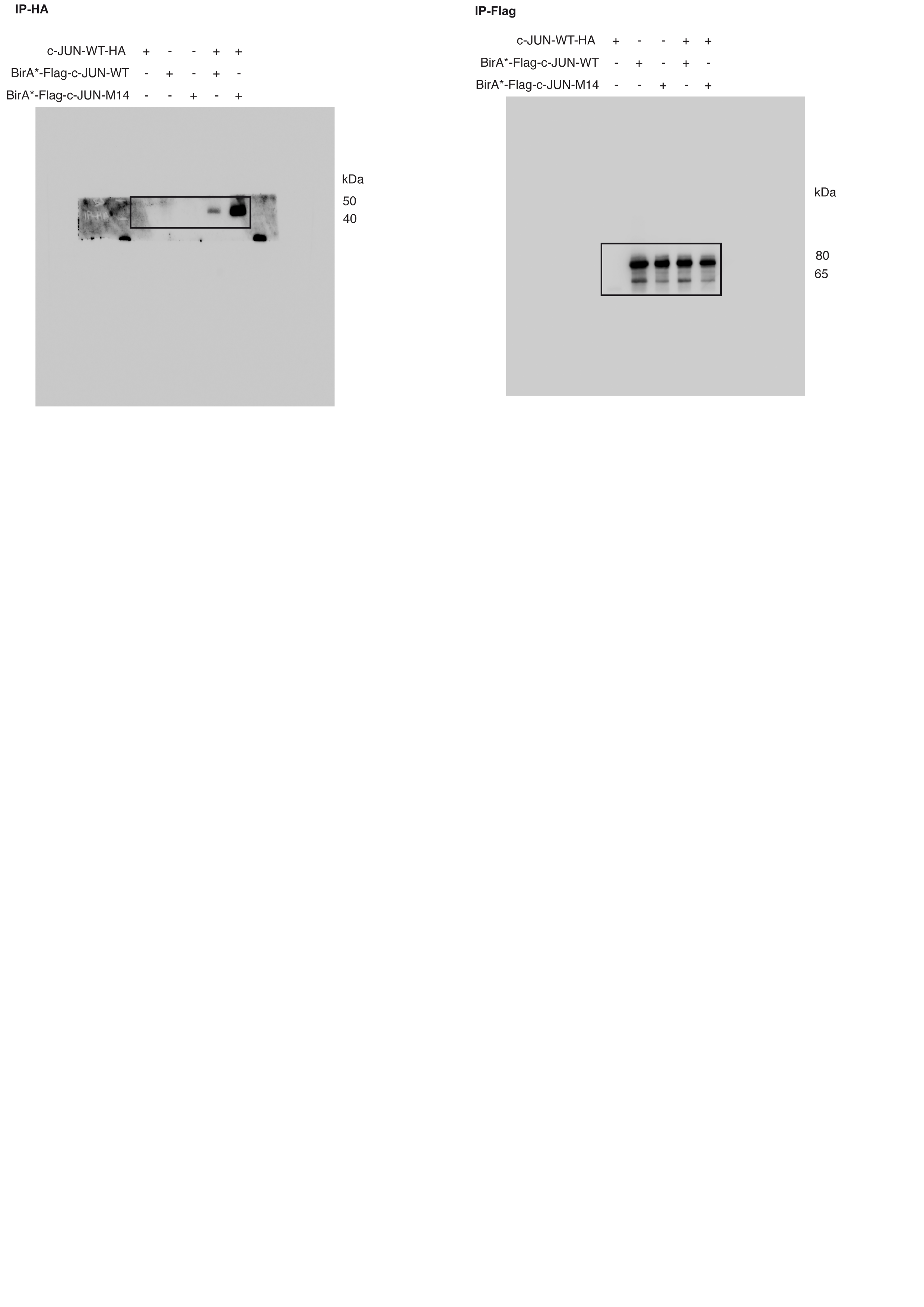

Supplement: Supplementary file 11 — Source data Fig. 5 [file 44318_2024_188_MOESM11_ESM.zip › Figure_5/5I/Figure_5I_IP.tif]

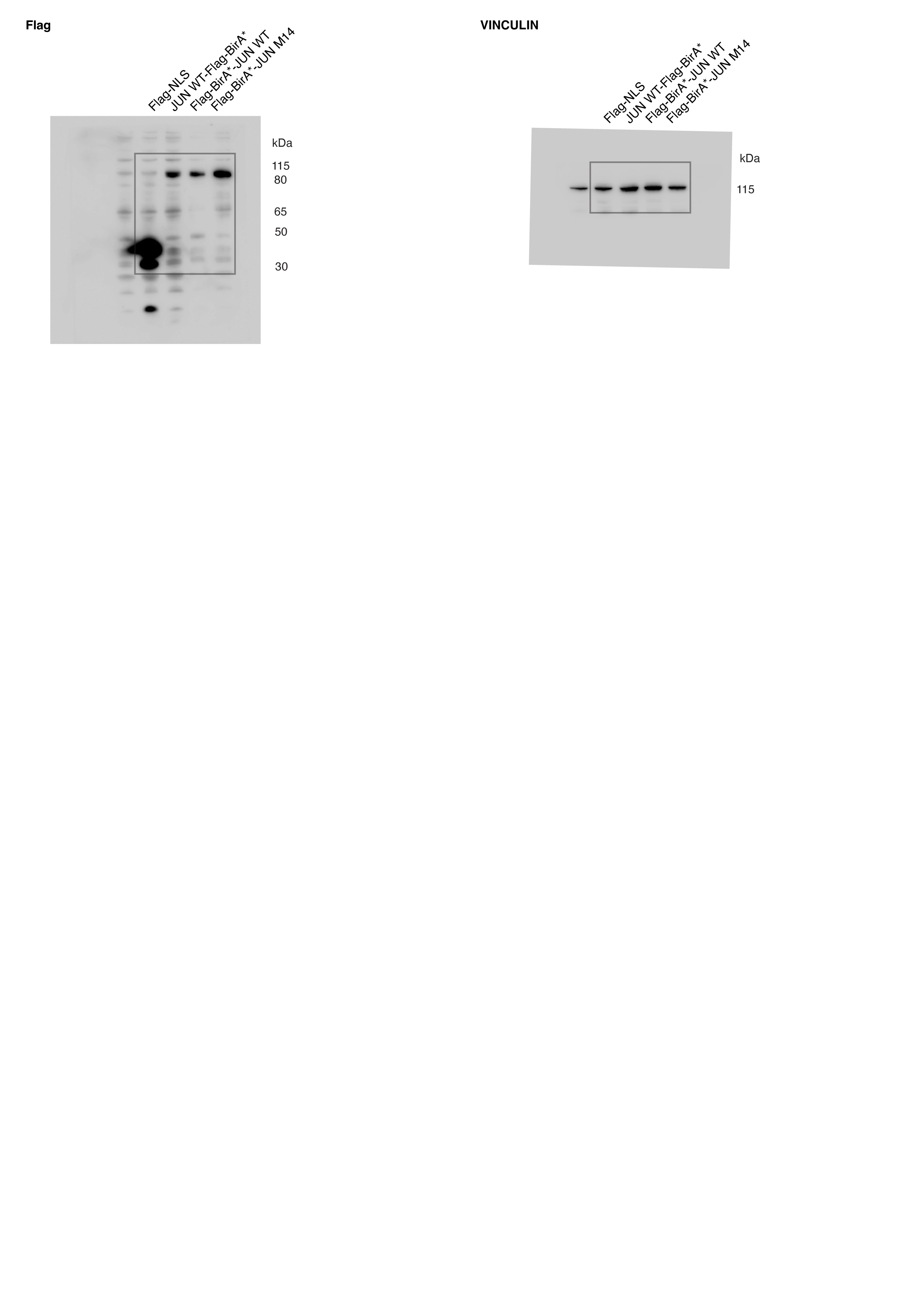

Supplement: Supplementary file 11 — Source data Fig. 5 [file 44318_2024_188_MOESM11_ESM.zip › Figure_5/5A/Figure_5A.tif]

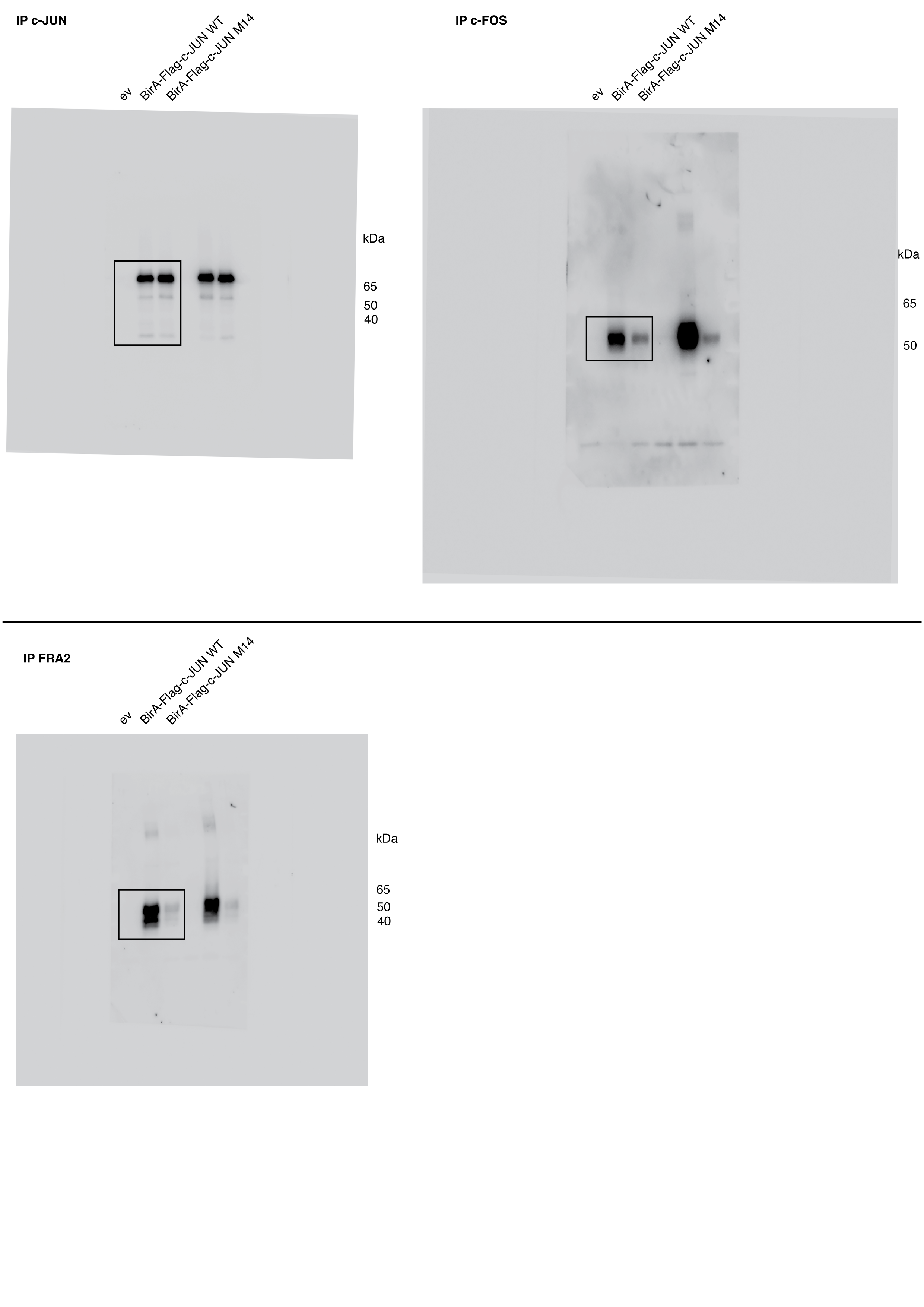

Supplement: Supplementary file 11 — Source data Fig. 5 [file 44318_2024_188_MOESM11_ESM.zip › Figure_5/5H/Figure_5H_IP.tif]

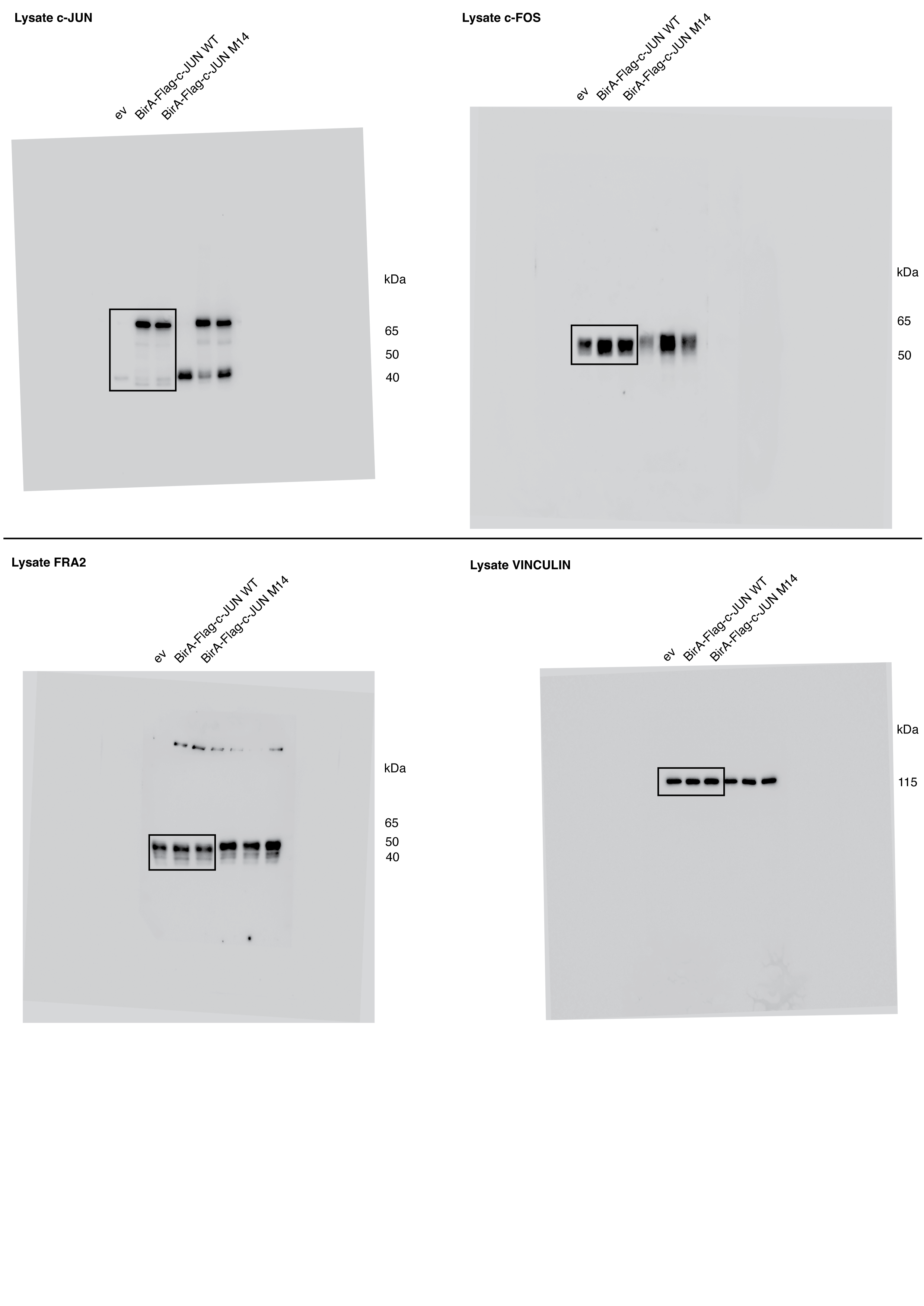

Supplement: Supplementary file 11 — Source data Fig. 5 [file 44318_2024_188_MOESM11_ESM.zip › Figure_5/5H/Figure_5H_Lysate.tif]

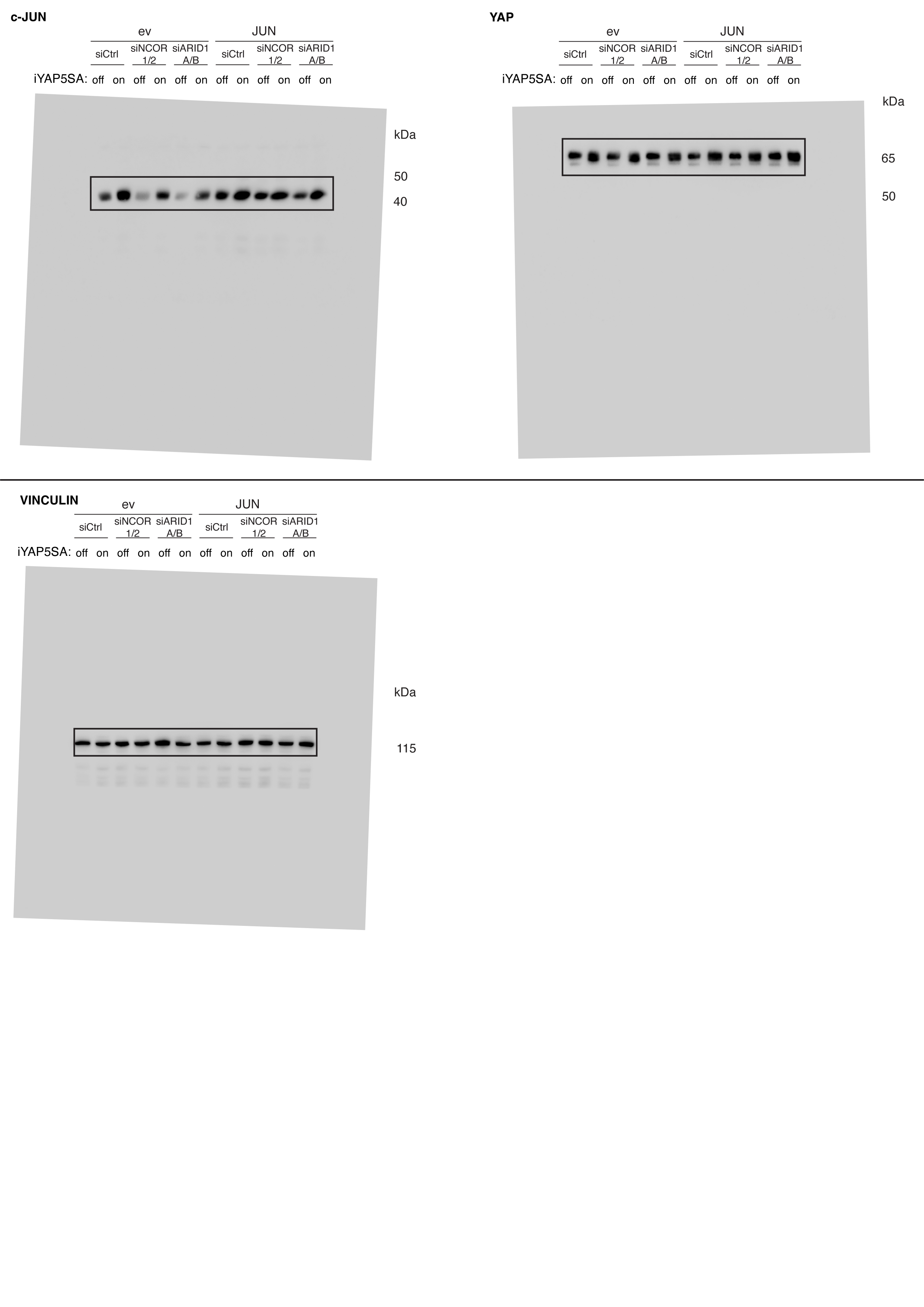

Supplement: Supplementary file 12 — Source data Fig. 6 [file 44318_2024_188_MOESM12_ESM.zip › Figure_6/6A/Figure_6A_JUN_YAP_VINCULIN.tif]

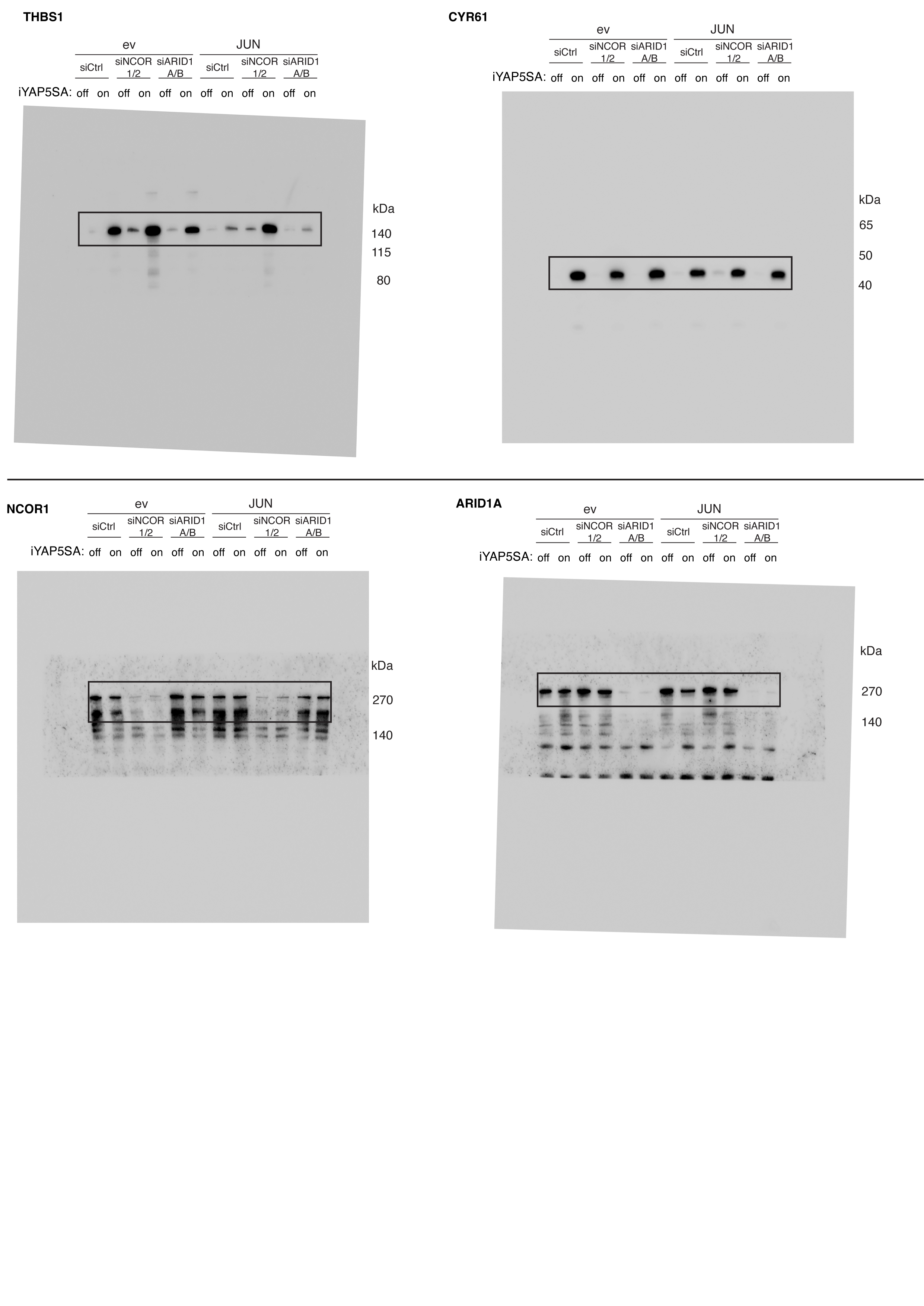

Supplement: Supplementary file 12 — Source data Fig. 6 [file 44318_2024_188_MOESM12_ESM.zip › Figure_6/6A/Figure_6A_THBS1_CYR61_NCOR1_ARID1A.tif]

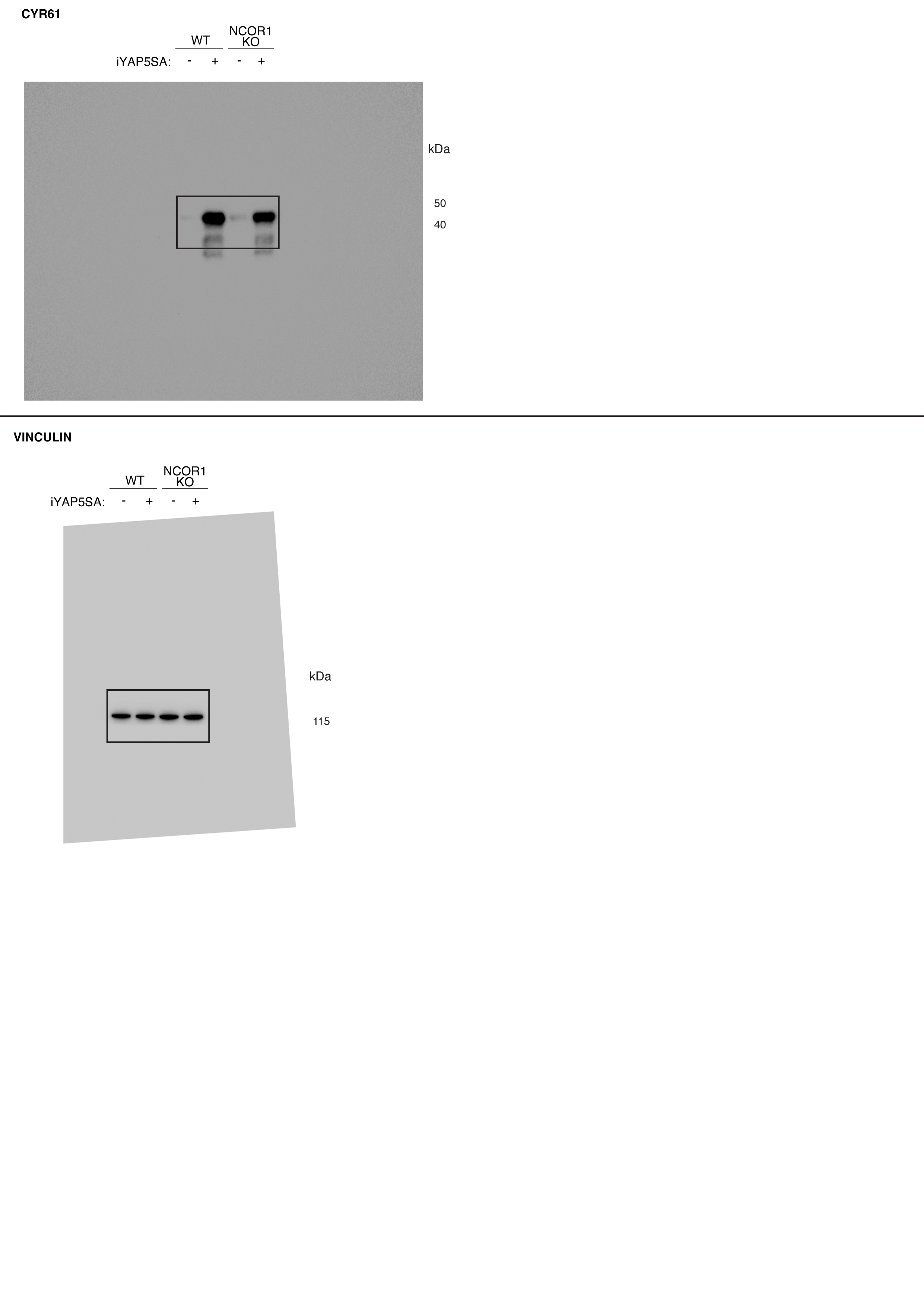

Supplement: Supplementary file 12 — Source data Fig. 6 [file 44318_2024_188_MOESM12_ESM.zip › Figure_6/6B/Figure_6B_CYR61_VINCULIN.tif]

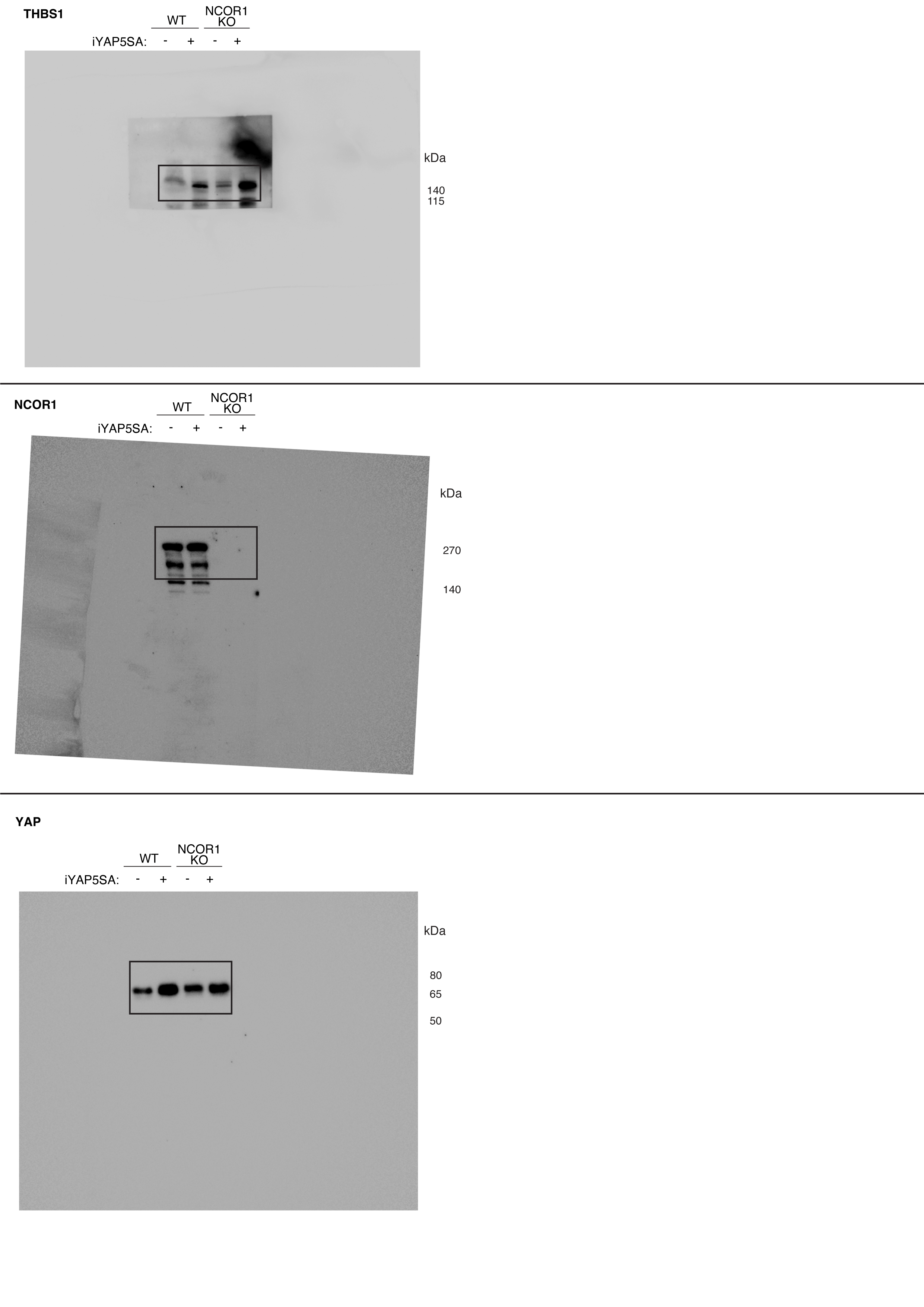

Supplement: Supplementary file 12 — Source data Fig. 6 [file 44318_2024_188_MOESM12_ESM.zip › Figure_6/6B/Figure_6B_THBS1_NCOR1_YAP.tif]

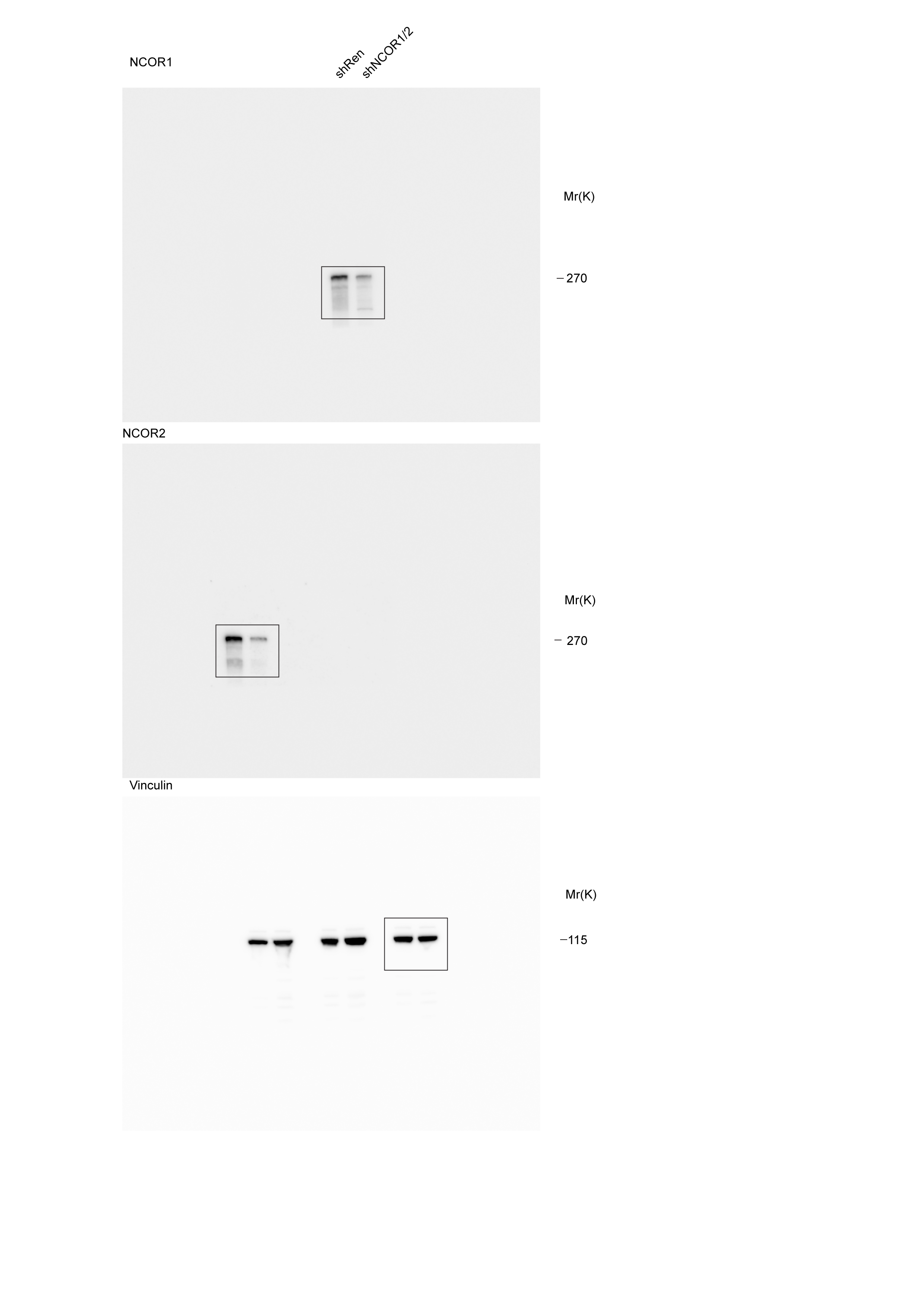

Supplement: Supplementary file 13 — Source data Fig. 7 [file 44318_2024_188_MOESM13_ESM.zip › Figure_7/7J/Figure_7J.tif]
