# Supplementary material for: A non-canonical repressor function of JUN restrains YAP activity and liver cancer growth
Source: EMBO J. 2024 Aug 29;43(20):4578–603. doi: 10.1038/s44318-024-00188-0 (PMC11480203; doi:10.1038/s44318-024-00188-0)
Supplement: Supplementary file 14 — Expanded View Figures [file 44318_2024_188_MOESM14_ESM.pdf]

Expanded View Figures

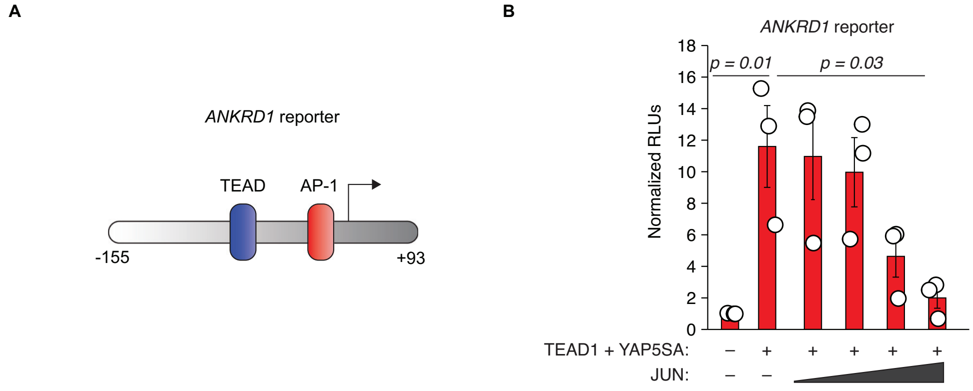

Figure EV1. JUN suppresses YAP-dependent induction of the ANKRD1 promoter.

(**A**) Schematic of the *ANKRD1* reporter construct used for the luciferase reporter assay. (**B**) Luciferase activity of the ANKRD1 reporter in 293T cells co-transfected with vectors for expression of HA-TEAD1 and FLAG-YAP5SA. Increasing amounts of JUN repress the reporter activity. Data shown are from three biological replicates. One-way ANOVA. RLUs = Relative luciferase light units. The error bars indicate the standard error of the mean.

 © The Author(s)

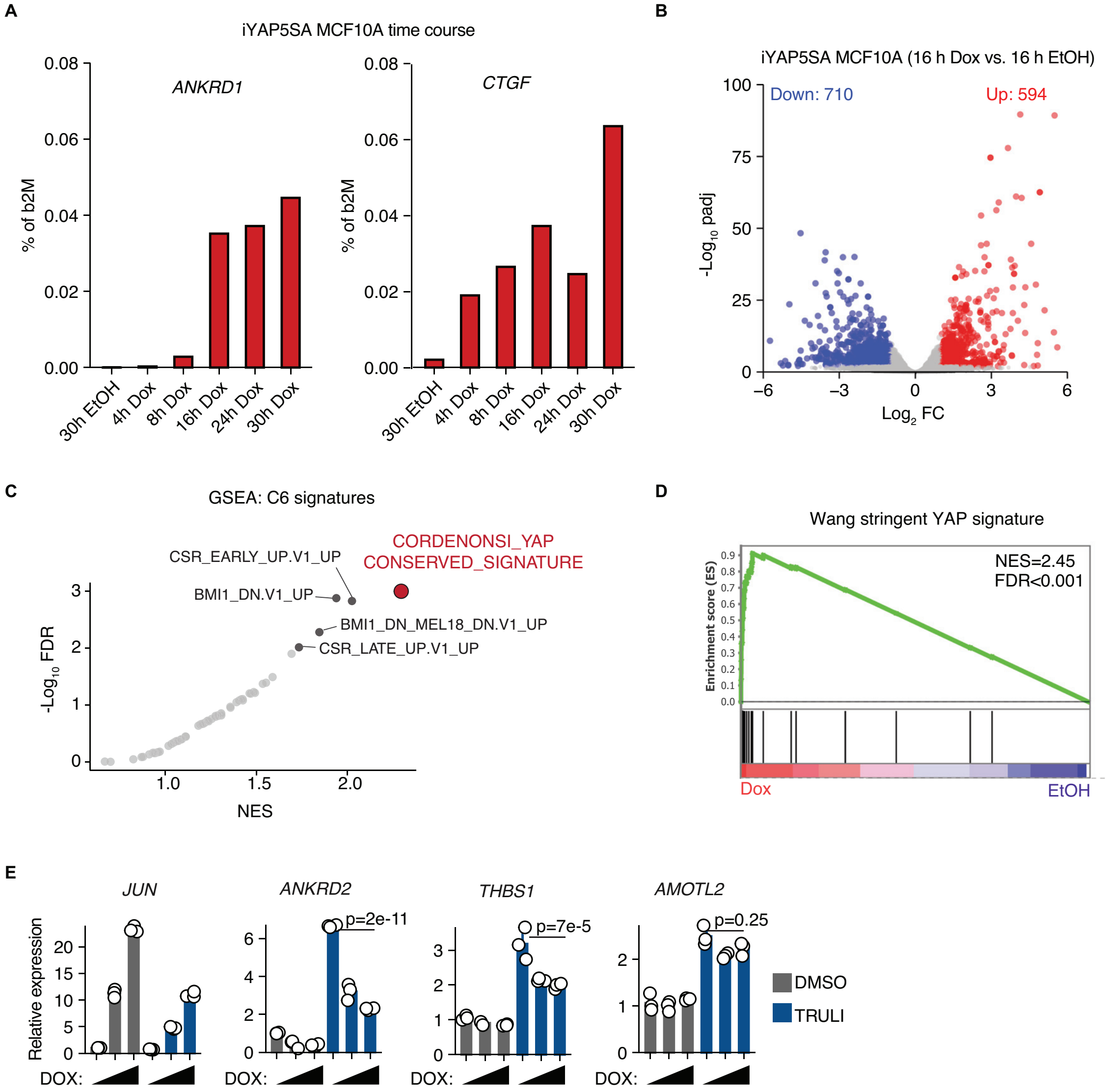

Figure EV2. The Hippo-insensitive YAP5SA allele induces expression of direct YAP target genes.

(**A**) qRT-PCR analysis of YAP target genes, *ANKRD1* and *CTGF*, in iYAP5SA MCF10A cells. YAP5SA expression was induced for indicated times with doxycycline or cells were treated with ethanol as a solvent control. Expression values are shown as percentage of b2M as housekeeping gene. Dox = doxycycline, EtOH = ethanol, $n = 1$. (**B**) Volcano plot showing differentially expressed genes upon YAP5SA expression in iYAP5SA MCF10A cells. The cells were treated with either doxycycline or ethanol for 16 h prior to RNA isolation and RNA-Sequencing library preparation. Significantly upregulated genes (determined by DESeq2) are highlighted in red and significantly downregulated genes are highlighted in blue. padj = adjusted *p*-value, FC = fold change. (**C**) GSEA summary of upregulated gene sets from RNA-Sequencing data upon YAP5SA induction in MCF10A-iYAP5SA cells ($n = 3$, biological replicates). NES = normalized enrichment score, FDR = false discovery rate. (**D**) GSEA enrichment plot for the Wang stringent YAP gene signature as a strongly upregulated gene set upon YAP5SA induction in MCF10A-iYAP5SA cells. NES = normalized enrichment score, FDR = false discovery rate. (**E**) qRT-PCR analysis of YAP target genes in MCF10A cells carrying a doxycycline-inducible JUN allele. JUN expression was induced by treating the cells with increasing concentrations of doxycycline (16.6 ng/ml and 50 ng/ml) or cells were incubated with ethanol as control. Expression of YAP target genes was induced using 10 μM LATS inhibitor TRULI. As control, cells were treated with DMSO. One-way ANOVA. DOX = doxycycline. The error bars indicate the standard error of the mean.

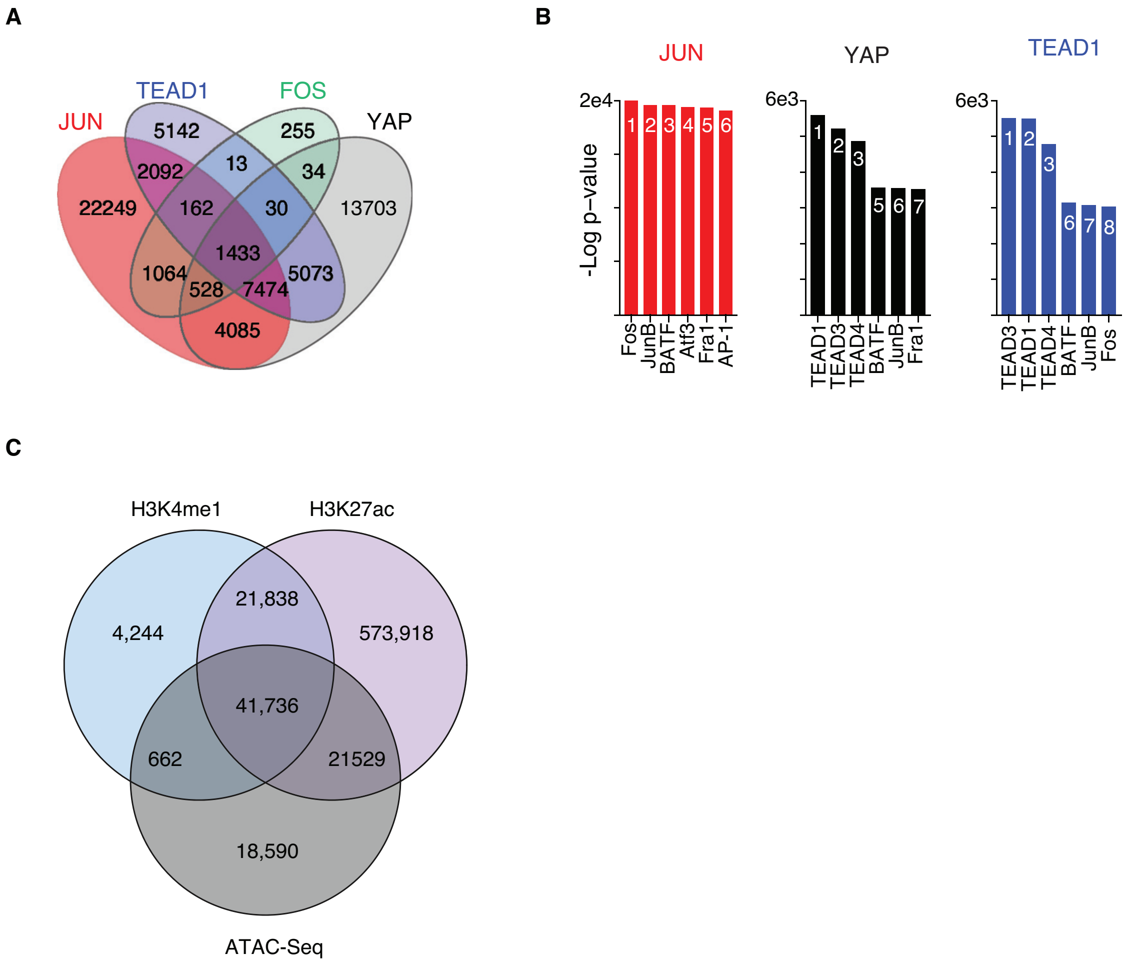

Figure EV3. JUN is recruited by YAP to genomic sites.

(**A**) Venn diagram for CUT&RUN peaks in YAP5SA-overexpressing MCF10A cells. (**B**) Motif enrichment analysis (by HOMER) for all CUT&RUN peaks from JUN, YAP, TEAD1. The numbers indicate the rank in the motif analysis. (**C**) Venn diagram for CUT&RUN enhancer marks (H3K4me1 and H3K27ac) and the ATAC-Seq signals.

© The Author(s)

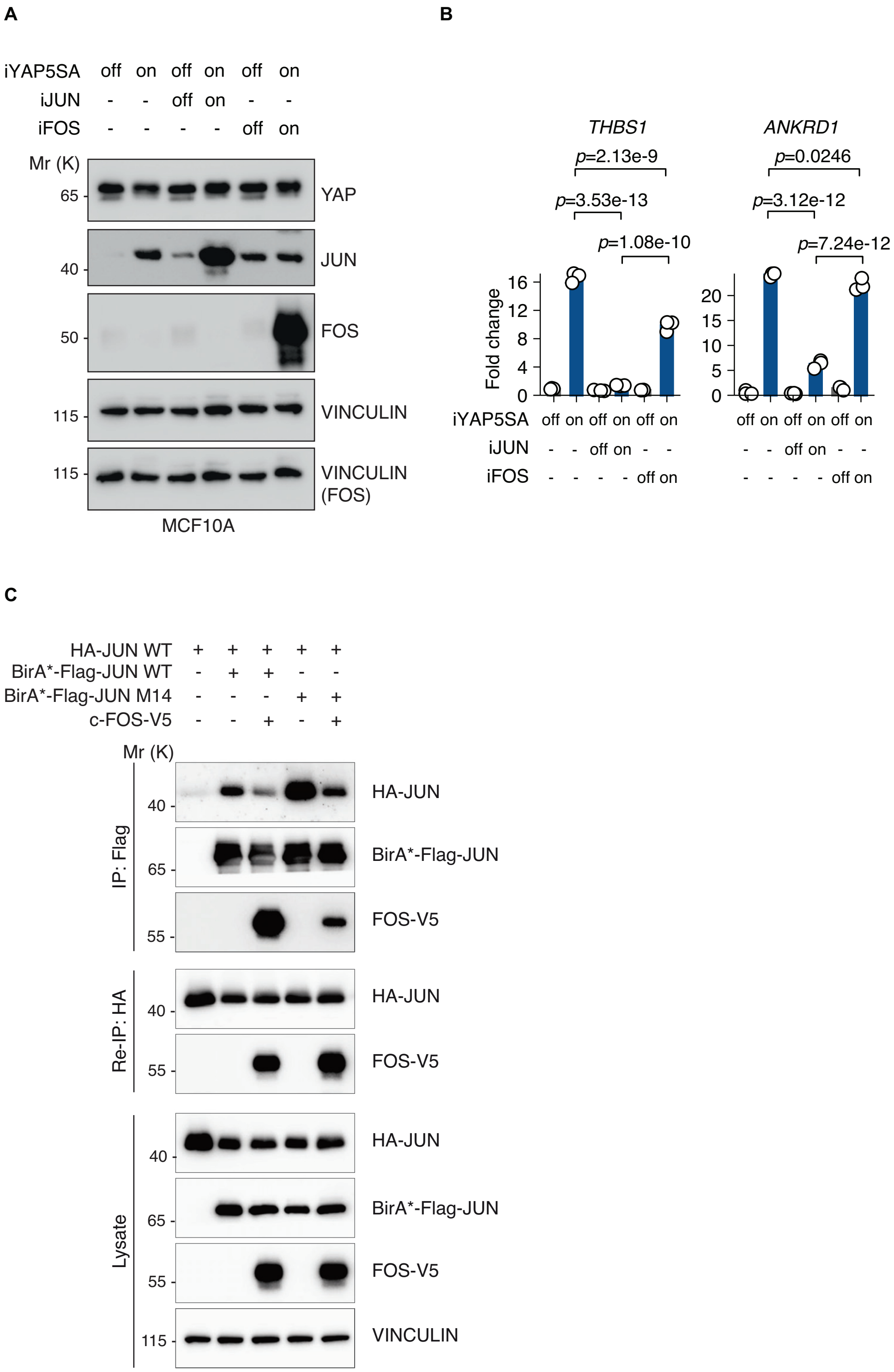

Figure EV4. JUN-mediated repression of YAP target genes is FOS-independent.

(**A**) Immunoblot from inducible MCF10A cells after induction of iYAP5SA, iJUN, and iFOS expression. The cells were treated with 100 ng/ml doxycycline for 20 h prior to analysis. As control, cells were incubated with the same volume of ethanol. VINCULIN was used as loading control, $n = 1$. (**B**) qRT-PCR analysis of the cells from (**A**) ($n = 3$, biological replicates). One-way ANOVA. The error bars indicate the standard error of the mean. (**C**) Co-immunoprecipitation experiments from 293T cells transiently transfected with the indicated constructs. BirA* fusion proteins were immunoprecipitated with Flag, and immunoprecipitates were assayed for HA-JUN and FOS-V5. After Flag precipitation, supernatants were further immunoprecipitated with HA and assayed for FOS-V5, $n = 2$ (biological replicates).

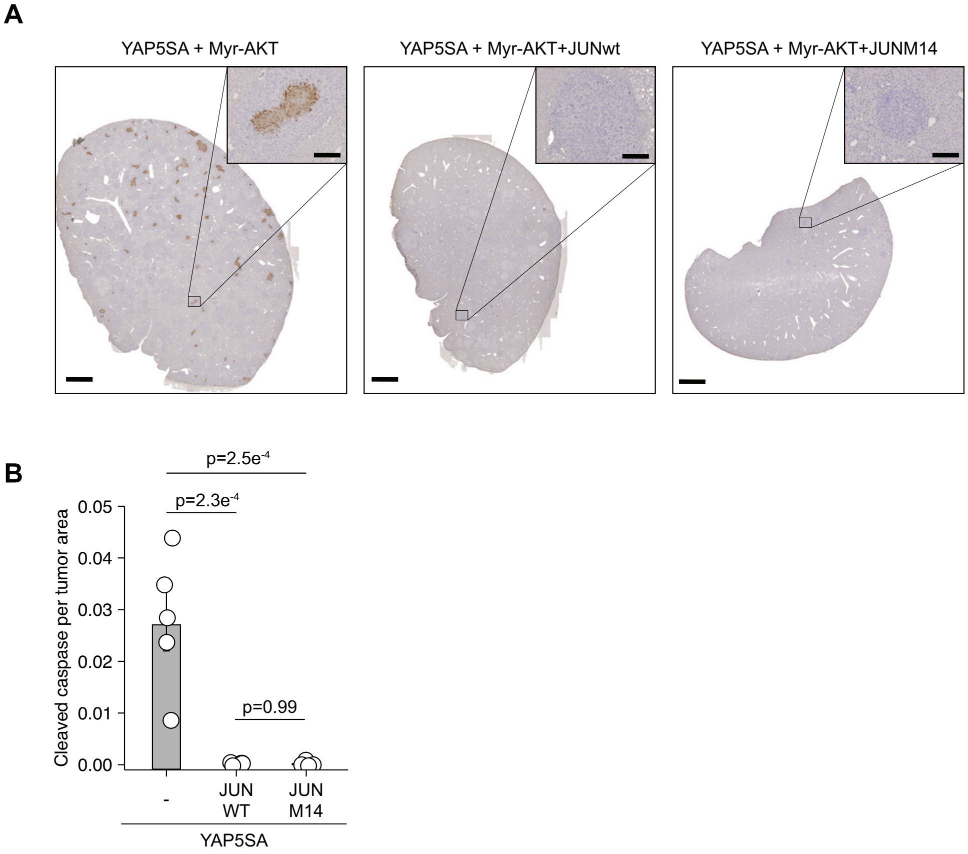

Figure EV5. JUN suppresses YAP-induced tumor growth, despite inhibiting apoptosis.

(**A**) Representative immunohistochemical staining of cleaved Caspase-3 on HDTVI liver sections. Scale bar = 2 mm. (**B**) Quantification of cleaved Caspase-3 signals in tumors for the indicated groups. $n = 5$ per experimental group. One-way ANOVA with Tukey HSD post hoc test. One-way ANOVA. The error bars indicate the standard error of the mean.

 © The Author(s)
